# Interactions of 2’-O-methyl oligoribonucleotides with the RNA models of the 30S subunit A-site

**DOI:** 10.1371/journal.pone.0191138

**Published:** 2018-01-19

**Authors:** Maciej Jasiński, Marta Kulik, Monika Wojciechowska, Ryszard Stolarski, Joanna Trylska

**Affiliations:** 1 Centre of New Technologies, University of Warsaw, Warsaw, Poland; 2 College of Inter-Faculty Individual Studies in Mathematics and Natural Sciences, University of Warsaw, Warsaw, Poland; 3 Department of Biophysics, Institute of Experimental Physics, Faculty of Physics, University of Warsaw, Warsaw, Poland; Weizmann Institute of Science, ISRAEL

## Abstract

Synthetic oligonucleotides targeting functional regions of the prokaryotic rRNA could be promising antimicrobial agents. Indeed, such oligonucleotides were proven to inhibit bacterial growth. 2’-O-methylated (2’-O-Me) oligoribonucleotides with a sequence complementary to the decoding site in 16S rRNA were reported as inhibitors of bacterial translation. However, the binding mode and structures of the formed complexes, as well as the level of selectivity of the oligonucleotides between the prokaryotic and eukaryotic target, were not determined. We have analyzed three 2’-O-Me oligoribonucleotides designed to hybridize with the models of the prokaryotic rRNA containing two neighboring aminoglycoside binding pockets. One pocket is the paromomycin/kanamycin binding site corresponding to the decoding site in the small ribosomal subunit and the other one is the close-by hygromycin B binding site whose dynamics has not been previously reported. Molecular dynamics (MD) simulations, as well as isothermal titration calorimetry, gel electrophoresis and spectroscopic studies have shown that the eukaryotic rRNA model is less conformationally stable (in terms of hydrogen bonds and stacking interactions) than the corresponding prokaryotic one. In MD simulations of the eukaryotic construct, the nucleotide U1498, which plays an important role in correct positioning of mRNA during translation, is flexible and spontaneously flips out into the solvent. In solution studies, the 2’-O-Me oligoribonucleotides did not interact with the double stranded rRNA models but all formed stable complexes with the single-stranded prokaryotic target. 2’-O-Me oligoribonucleotides with one and two mismatches bound less tightly to the eukaryotic target. This shows that at least three mismatches between the 2’-O-Me oligoribonucleotide and eukaryotic rRNA are required to ensure target selectivity. The results also suggest that, in the ribosome environment, the strand invasion is the preferred binding mode of 2’-O-Me oligoribonucleotides targeting the aminoglycoside binding sites in 16S rRNA.

## Introduction

**Fig 1:**
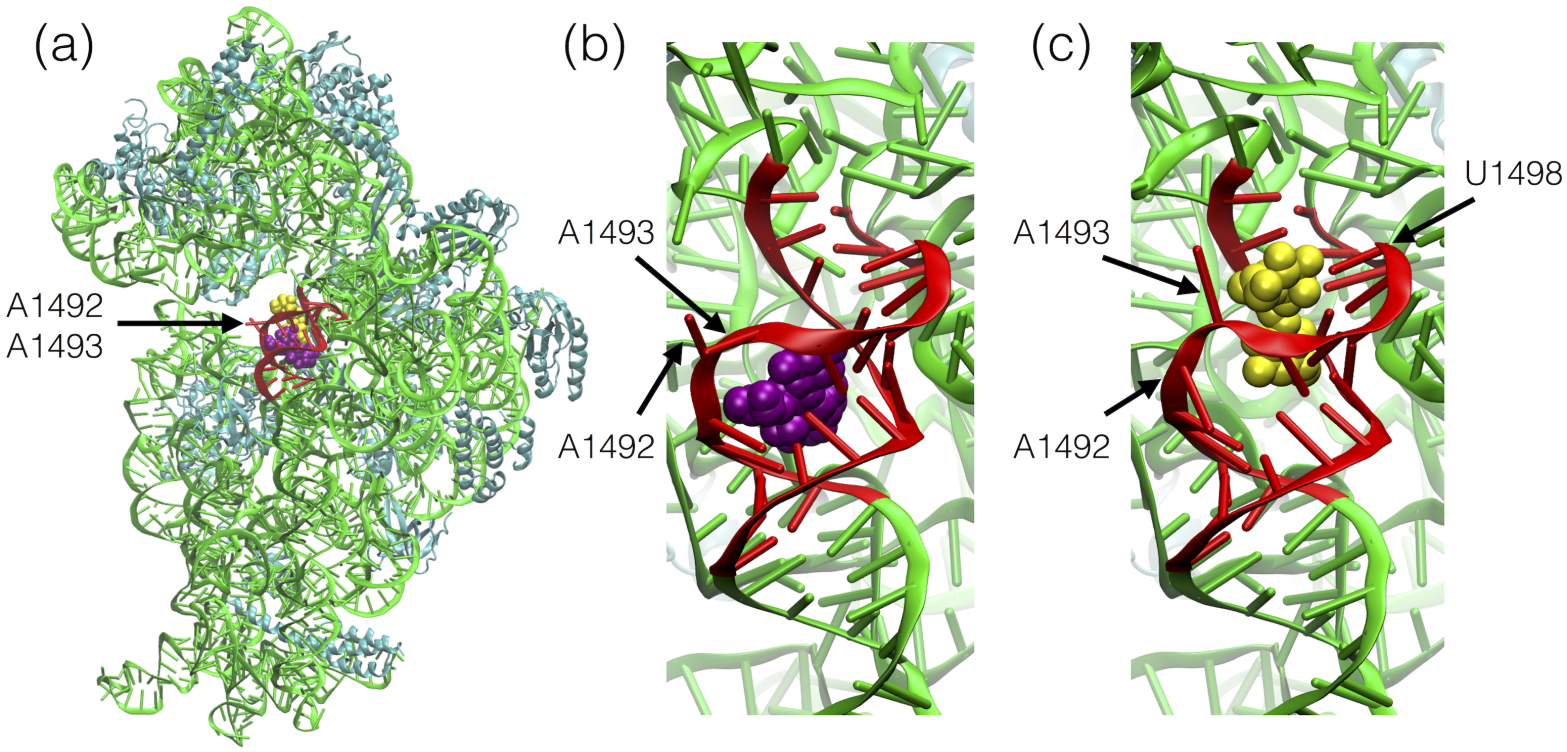
Paromomycin (purple) and hygromycin B (yellow) in their primary binding sites in the rRNA helix h44 of the 30S subunit of the bacterial ribosomes. RNA is in green and proteins in cyan. Red denotes the rRNA fragment included in the studied model of the prokaryotic rRNA (PDB code: 3LOA [6]). (a) The position of the antibiotics in the 30S subunit. (b) Zoom of paromomycin binding site (PDB code: 2Z4K [7]). (c) Zoom of hygromycin B binding site (PDB code: 3DF3 [8]).

The ribosomes, composed of rRNA and proteins, catalyze polypeptide synthesis in living cells. They are built up of two subunits, small and large, which in prokaryotic ribosomes are called 30S and 50S. There are three tRNA binding sites (denoted as A, P, and E) at the interface between the subunits. The aminoacyl-tRNA binding site (A-site) in helix h44 of 16S rRNA is responsible for verifying the mRNA codon tRNA-anticodon complementarity. The adenines 1492 and 1493 (according to the *E. coli* rRNA numbering) in helix 44 (Fig 1a) comprise a molecular switch in the ribosome that controls the fidelity of the mRNA encoding [1, 2]. When flipped-out, in the so-called active state, the adenines form a complex with the anticodon of the cognate tRNA. In the inactive state, they are in a slightly energetically preferred intra-helical conformation [3] and the non-cognate tRNA cannot be accepted in the A-site [4]. This functionally important region of 16S rRNA overlaps also with the inter-subunit contact, termed the B2a bridge, which is formed between the penultimate stem of helix h44 of 16S rRNA and helix 69 of 23S rRNA of the large subunit [5].

The bacterial ribosome, due to its crucial function in translation, is a target for many antibiotics [9, 10]. The A-site in the 30S subunit is a primary binding site for 2-deoxystreptamine (2-DOS) aminoglycosides [11]. The 2-DOS aminoglycosides, such as neomycin, paromomycin, kanamycin or gentamicin affect the fidelity of translation by locking A1492 and A1493 in a flipped-out state (Fig 1b) which promotes decoding errors by allowing incorporation of near-cognate and non-cognate tRNAs [12].

Hygromycin B, another 2-DOS containing aminoglycoside, that binds near the A-site (Fig 1c), has a different binding mode that affects the translocation of mRNA and tRNAs during polypeptide elongation [8]. The universally conserved U1498 is one of the nucleotides that makes base-specific hydrogen bonds with hygromycin B [13]. Moreover, U1498 helps position mRNA in the 30S subunit P-site by making a number of contacts to the backbone of nucleotides +1 and +2 of the mRNA [14], as well as plays an active role in the translocation of mRNA and tRNAs through the ribosome [15]. In addition, the U1498C mutation is known to cause weak resistance to hygromycin B in *M. smegmatis* [16] but the molecular mechanism of this resistance remains unclear [8].

Due to development of bacterial resistance to existing antibiotics, possible antibiotic toxicity and insufficient specificity toward pathogenic bacteria, there is a need to discover or develop new antimicrobials. Efforts to modify aminoglycosides or find new scaffolds had only few successes [17–19]. Other ways included designing peptides that specifically bound the 30S subunit A-site and inhibited translation *in vitro* [20]. However, natural peptides are degraded by proteases and may have problems with solubility [21]. Another approach could be to use oligonucleotides that bind in the sequence-specific manner and sterically block a functional rRNA fragment leading to inhibition of bacterial translation [22]. Such oligonucleotides would be advantageous because their sequence could be easily redesigned in response to bacterial mutations that cause resistance to known antibiotics. Indeed, modified oligonucleotides such as peptide nucleic acids (PNA) and 2’-O-methylated oligoribonucleotides (2’-O-Me RNA) were used to determine their hybridization efficiency with a 16S rRNA fragment [23, 24]. Overall, at pH 5.5, PNA and guanidine-modified PNA, had modest binding affinity and low sequence selectivity, with the binding order indicating a PNA_2_-RNA triplex. Interestingly, the 3-oxo-2,3-dihydropyridazine- modified PNA exhibited micromolar binding affinity for the bacterial 16S RNA A-site model at pH 6.25, while no binding was observed to the model of the human A-site [25]. The binding stoichiometry close to 1:1 suggested the formation of the PNA-RNA_2_ helix.

Interactions of 2’-O-Me oligonucleotides with the A-site rRNA were conducted also in the context of the whole 30S ribosomal subunit. Abelian et al. [26] targeted the A-site with twelve, overlapping 10-mer 2’-O-Me RNAs, complementary to the region U1485—G1516 of 16S RNA. The oligomer that targeted the A1493—A1502 fragment showed the lowest *K*_*d*_ of 29 *nM* for the binding to the 30S subunit. Interestingly, the oligomers with nucleotides complementary to A1492 and/or A1493 showed dose-dependent increase in binding upon adding paromomycin. Finally, it was shown that these 2’-O-Me RNAs inhibit translation *in vitro* and that there is some limited correlation between oligomer inhibitory activity and its binding affinity to the A-site. We have also verified the binding of a 2’-O-Me RNA oligomer complementary to the A1493–A1502 16S RNA sequence to the 30S subunit and 70S ribosomes, its anti-translational activity in the cell-free system and in addition, have shown that after transformation into *E. coli* cells this oligomer inhibited their growth [27].

However, in the above studies structural details of the hybridized complexes were not provided. In principle, there are two possible binding modes: strand invasion via Watson-Crick base pairing and triplex formation via Hoogsteen-type interactions. In this work we examined the interactions of three 2’-O-Me oligoribonucleotides with the models of the 16S rRNA decoding A-site. As a model of the prokaryotic A-site rRNA we used a bipartite system, which covers two aminoglycoside binding sites. This system was reported as a faithful target to monitor ligand binding to the ribosomal decoding site [6]. We designed a structurally comparable model of the eukaryotic decoding A-site by introducing six mutations to the above prokaryotic model. The objective was to determine if the 2’-O-Me oligomers bind to the prokaryotic 16S rRNA A-site model but at the same time do not form a stable complex with the eukaryotic model. We also examined if there is any relation between the flexibility of the A-site models determined from MD simulations and melting temperatures determined in solution studies of these constructs. With MD simulations we characterised for the first time the dynamics of the hygromycin B binding site in the models of both prokaryotic and eukaryotic rRNA. This was not possible in our previous studies that were performed using smaller A-site models covering only the closest neighbourhood of A1492 and A1493 [28, 29].

## Materials and methods

### Reagents

**Fig 2:**
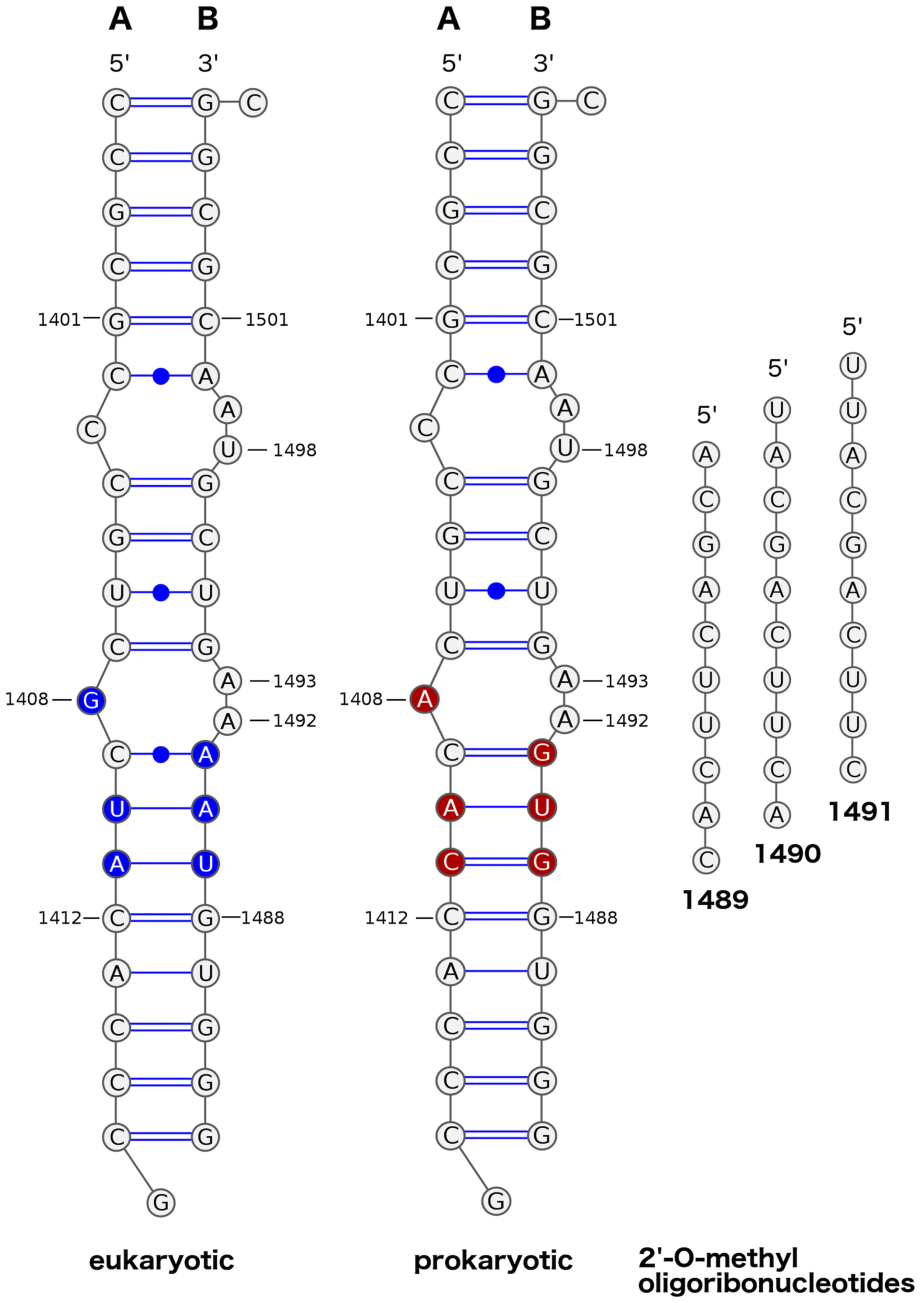
Secondary structures of the eukaryotic and prokaryotic targets and 2’-O-Me oligoribonucleotides. Numbering of all nucleotides is as in the *E. coli* ribosome. Nucleotides coloured in the target structures are specific for the *H. sapiens* and *E. coli* ribosomes. Nucleotides in the base pair range of 1401–1501 and 1412–1488 are identical as in the small ribosomal subunits of relevant organisms. Four additional base pairs at each terminus of the model were added, the same as in the work of Dibrov et al. [6] to stabilize the termini.

Gel-purified synthetic RNA oligonucleotides shown in Fig 2 (eukaryotic A: 5’-CCG CGC CCG UCG CUA CAC CCG-3’; eukaryotic B: 5’-GGG UGU AAA AGU CGU AAC GCG GC-3’; prokaryotic A: 5’-CCG CGC CCG UCA CAC CAC CCG-3’; prokaryotic B: 5’-GGG UGG UGA AGU CGU AAC GCG GC-3’) as well as 2’-O-methylated oligoribonucleotides (1489: 5’-ACG ACU UCA C-3’; 1490: 5’-UAC GAC UUC A-3’; 1491: 5’-UUA CGA CUU C-3’) and 2-amino purine (2AP) labeled RNA were purchased from Future Synthesis, Poland. 2AP was placed in the position of A1493 in the strand B of both models.

### UV melting

UV absorbance profiles as a function of temperature were measured at 260 nm using the UV-Vis Nicolet Evolution 300 Spectrophotometer (Thermo Scientific). 2’-O-Me oligonucleotides were mixed at 2 μM concentration of each strand in 10 mM phosphate buffer with 20 mM NaCl, pH 7.0. Samples were heated from 10°C to 90°C at a rate of 1°C/min. In the case of two-state transitions, the absorbance versus temperature curves were fitted to a two state model with sloping baselines [30], from which the fraction of the base paired molecules (*f*) versus temperature (T) was calculated. The melting temperature (*T*_*m*_) was defined as the temperature for which *f*(*T*) = 0.5 [30, 31]. From *f*(*T*), the temperature dependent *K*_*a*_ values were calculated, using the equation appropriate for non-self-complementary complexes [31]:

$$\begin{array}{c} K_{a}=\frac{2f}{{\left(1-f\right)}^{2}c_{0}} \end{array}$$ (1)

where *c*_0_ is the concentration of a single strand. Free energy of the duplex formation (Δ*G*_*UV*_) was obtained by fitting the linear function to the van’t Hoff plot [30]:

$$\begin{array}{c} \ln K_{a}=-\frac{\Delta H}{R}\frac{1}{T}+\frac{\Delta S}{R} \end{array}$$ (2)

where *R* is the ideal gas constant and Δ*H* is the enthalpy change independent of temperature that corresponds to T = 21°C. The nonlinear fitting that took into account the temperature dependence of Δ*H*, i. e. assuming nonzero values of the heat capacity changes Δ*c*_*p*_ [32], provided similar results.

### Fluorescence spectroscopy

Fluorescence measurements were performed on a thermostatted Hitachi F-7000 fluorescence spectrophotometer at 21°C. Emission spectra were recorded in the 10 mM phosphate buffer with 20 mM NaCl, pH 7.0 while exciting at 310 nm. Normalized relative fluorescence was calculated by subtracting the background signal measured as the titration of the buffer to the compound and normalization by the fluorescence intensity of the free labeled RNA.

### Isothermal titration calorimetry

ITC measurements were performed at 21°C using Low Volume Nano ITC equipment (TA instruments) in 10 mM phosphate buffer with 20 mM NaCl, pH 7.0. In the experiments evaluating the stability of the 16S rRNA A-site models, twenty 2 μL aliquots of the A strand with the concentration of 100 μM were injected into the solution of the B strand with the concentration of 10 μM. In the studies of the interactions of 2’-O-Me oligomers with rRNA targets, the 2’-O-Me oligomers with concentration of 70 μM were injected in twenty five 2 μL aliquots into the 10 μM solution of the prokaryotic rRNA B strand. Time between injections (3 to 10 minutes) was adjusted to ensure the equilibrium after every injection. The injections were continued past saturation to measure the heat related to dilution and the average calculated dilution heat was subtracted from the heats released upon titration. The peak areas were integrated by the Nano Analyzer software provided with the instrument. Standard uncertainties were determined from imprecisions of the model fitting to the experimental data. ITC provides direct measurements of the enthalpy changes (Δ*H*) of the interaction between molecules and the binding affinity (*K*_*a*_) and stoichiometry (*n*) can be determined from the fitting. Further, from these measurements the changes in Gibbs energy (Δ*G*_*ITC*_) T = 21°C and entropy (Δ*S*) were obtained using the relationship [33]:

$$\begin{array}{c} \Delta G_{ITC}=-RT\ln K_{a}=\Delta H-T\Delta S \end{array}$$ (3)

### Gel electrophoresis

Polyacrylamide gel electrophoresis was performed under non-denaturing conditions using a 15% (19:1) acrylamide matrix and a solution of 1xTBE (89 mM Tris-base, 89 mM borate, 2 mM EDTA, pH 8.3) as the running buffer. RNA and 2’-O-Me RNA oligomers in the 400 pmol concentration were incubated in the buffer consisting of 100 mM NaCl, 20 mM MES and 0.5 mM EDTA at pH 5.5 for 1 h before loading. Then 1 μl of 40% glycerol was added into 5 μl of samples. The gels were run at 80 V for 3 h and then were stained by Stains-All (E9379, Sigma-Aldrich). The resulting bands were imaged by a Gel Doc XR+ System (Bio-Rad).

### Molecular dynamics

#### Force field parameters and simulation conditions

MD simulations of prokaryotic and eukaryotic rRNA models were performed using NAMD [34] and bsc0*χ*_*OL*3_ version of the Amber force field [35–37]. Simulations were conducted in the NpT ensemble at 310 K and pressure of 1 bar, maintained with the Langevin algorithms [38, 39]. 310 K was selected as a standard temperature in MD simulations to achieve some physically reliable mobility of nucleotides since lower simulation temperature such as 295 K would be impractical in case of a flexible RNA system. Application of the SHAKE [40] algorithm for bonds involving hydrogens allowed us to use a 2 fs integration step. Periodic boundary conditions were applied. For evaluation of long-range interactions, the particle mesh Ewald method [41] was employed.

#### System construction and starting conformations

As a starting configuration of the prokaryotic A-site model, we used the crystal structure determined by Dibrov et al. [6] (PDB ID 3LOA). The eukaryotic model was prepared by introducing the following mutations: A1408G, A141U, C1411A, G1489U, U1490A, G1491A (Fig 2), as in the study describing the engineering of the functional human–bacterial hybrid ribosomes [42]. Thus, the initial conformation of A1492 and A1493 in the eukaryotic system was identical as in the prokaryotic system: A1492 occupied the extra-helical state and A1493 the intra-helical state.

Each model was immersed in a cubic box containing TIP3P [43] water molecules providing at least 20 Å layer of the solvent at each side of RNA strands. The negatively charged RNA were neutralized by adding Na^+^ ions, by replacing water molecules at positions of local minima of the electrostatic potential.

No additional ionic strength was used. Adding the minimum number of monovalent ions (enough to neutralize the system) is a standard way to reproduce well structural and dynamic characteristics of nucleic acids, regardless of the ion model used [44]. What is more, the method used to set the initial distribution of ions around nucleic acid models does not affect the simulation results. For monovalent ions, such as used here Na^+^, the ion distribution around nucleic acids obtained in MD simulations was found independent on the initial positions of ions [45]. The topology and coordinate input files were prepared using the tleap program from the Amber9 [46] package.

#### Simulation protocol

The simulation protocol, was adapted from our previous MD studies of nucleic acids [28, 47, 48] by extending the simulation time for each equilibration phase ten times. In short, the protocol began from equilibration of water molecules and ions, performed in the NVT ensemble and consisted of two substages. During the first 560 ps, the temperature was linearly increased from 30 to 310 K with harmonic restraints of 50 *kcal* · *mol*^−1^ *Å*^−2^ applied to all heavy atoms of RNA. Then, the restraints were relaxed to 25 *kcal* · *mol*^−1^ *Å*^−2^, and the simulation continued for 350 ps. During the next phase, 3 ns of equilibration in the NpT ensemble, the restraints were gradually decreased starting from 5 *kcal* · *mol*^−1^ *Å*^−2^ to close to 0, by reducing them by half after each 500 ps. The final equilibration phase (NpT) lasted 2 ns and was followed by a 1150 ns production stage for each system.

#### Data analysis

Trajectories were analyzed with the MINT package [49], ptraj module from AmberTools 1.5 [46] and in-house written Python scripts employing MDAnalysis library [50]. The criteria for the presence of hydrogen bonds were: 3.5 Å for the maximal acceptor-donor distance and minimal acceptor-hydrogen-donor angle was set to 150 degrees. The base pairs denoted as WC-WC are pairs in which both nucleotides interact via the Watson-Crick edge, which means that these pairs do not necessarily have to be the canonical A:U, G:C pairs [51]. Accordingly, the non-WC-WC base pairs are pairs in which at least one of the nucleotides interacts with the edge other than the Watson-Crick edge. The energy of the stacking interaction between nucleotides was estimated as the sum of Coulomb and van der Waals (VdW) interaction between all heavy atoms in interacting nucleobases.

Flipping of nucleotides A1492, A1493 and U1498 was monitored with the pseudo-dihedral angle introduced by Song et al. [52] and previously successfully applied in the studies investigating the decoding A-site dynamics [2, 29]. The four points defining the angle are: i) centre of mass of the flipping nucleobase, ii) centre of mass of the flipping nucleotide phosphate, iii) centre of mass of the phosphate of the next nucleotide and iv) centre of mass of the base pairs flanking analysed nucleotide. If the nucleobase is flipped-in, the angle is roughly in the range (-50°, +50°), and if the nucleobase is flipped-out the angle is close to ±180°. The value of the angle allows also estimating the orientation of nucleotides. In the used convention, if a nucleotide faces towards the major groove, the angle is from -180 to 0°, and if it faces towards the minor groove, the angle is in 0°– 180°range.

MD structures were subjected to the RMSD (root mean square deviation) based clustering using the gromos method [53] from the Gromos package [54]. The algorithm divides the set of conformations into separate clusters according to the RMSD criterium. VARNA [55] was used to produce secondary structure figures, VMD [56] and Chimera [57] to visualize trajectories and produce 3D structure images.

## Results

### Selection of rRNA models and 2’-O-methylated oligoribonucleotides

The model of the prokaryotic rRNA used in this study (PDB ID: 3LOA [6]) contains two overlapping aminoglycoside binding sites [16]: (i) the 4’-5’ and 4’-6’-2-DOS aminoglycoside (such as neomycin or kanamycin) binding site with nucleotides: C1407, A1408, G1491, A1492, A1493, G1494, U1495, and (ii) hygromycin B binding site composed of: C1403, C1404, 1405, G1494, U1495, C1496, G1497, U1498 (Fig 2). Previous solution studies confirmed that this construct with a fluorescent 2-amino purine (2AP) modification in the position of A1493, provides a model system that can be used to monitor ligand binding to the ribosomal decoding site [6]. The model of the eukaryotic A-site was prepared by introducing six mutations to the original prokaryotic construct (Fig 2) and was not previously reported. Therefore, to verify the eukaryotic construct, we first investigated its properties in solution in a similar way as for the previously reported prokaryotic system [6] but with the addition of ITC experiments. Three 2’-O-Me RNA decamers were selected based on the set of sequences that efficiently bind to bacterial ribosomes and inhibit translation in bacterial cell free systems [26]. Although, each of the 2’-O-Me oligonucleotides is fully complementary to the prokaryotic A-site, they have one, two or three mismatches to the eukaryotic model.

### Solution studies of the eukaryotic and prokaryotic decoding A-site rRNA models

**Fig 3:**
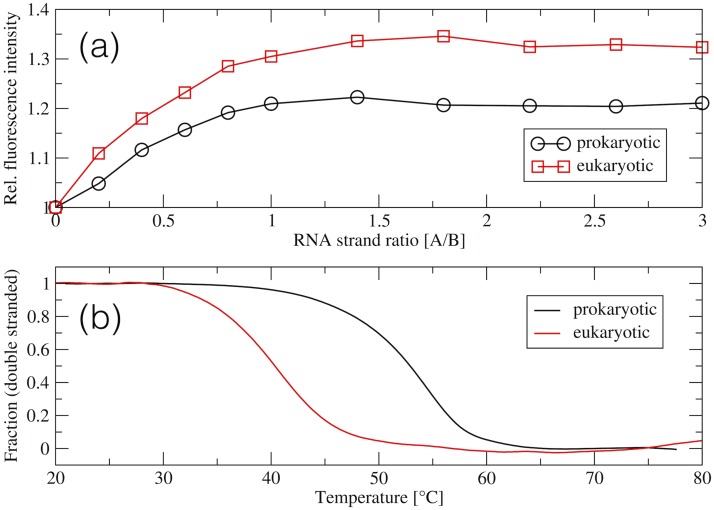
Solution studies of rRNA models. (a) 2AP fluorescence intensity during titration of the 2AP-labeled strand B (at the 1493 position) to strand A. (b) UV-melting profiles of the prokaryotic (*T*_*m*_ = 52.9°C) and eukaryotic (*T*_*m*_ = 42.6°C) decoding A-site models presented as the fraction of the double stranded structures versus temperature.

Using fluorescence spectroscopy we checked if the A and B strands of the eukaryotic model form a bipartite system (Fig 2). Titration of the 2AP1493 labeled strand B with the increasing amounts of the complementary strand A resulted in a steady increase of 2AP fluorescence intensity up to the 1:1 strand ratio (Fig 3a), which indicates full hybridization. Further, fluorescence intensities as a function of the RNA strand ratio, increased up to 20% in the prokaryotic and up to 30% in the eukaryotic model.

Thermal denaturation curves of Fig 3b show biphasic transitions for both models. From these melting curves, we also derived thermodynamic parameters (Table 1). Nucleotide substitutions incorporated to the prokaryotic model decreased the melting temperature by about 10°C.

**Table 1:** Thermodynamic parameters of the RNA strands forming decoding A-site models, derived from the UV melting profiles.

Model:	Δ*H* [kcal/mol]	TΔ*S* [kcal/mol]	Δ*G* [kcal/mol]	*T*_*m*_ [°C]
prokaryotic	-99.8 (0.9)	-81.5 (0.9)	-18.3 (1.8)	52.9 (0.5)
eukaryotic	-90.2 (0.7)	-76.1 (0.8)	-14.1 (1.5)	42.6 (0.5)

Δ*G* and TΔ*S* values calculated for T = 21°C. Values in parentheses represent standard errors from 3 independent experiments.

**Fig 4:**
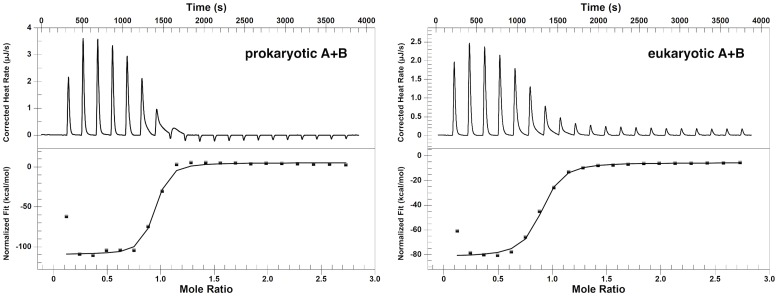
ITC scans of the titration of the A strand to the B strand of the prokaryotic and eukaryotic A-site models at T = 21°C.

Thermodynamic parameters for the interactions between the RNA oligomers were also determined with the ITC technique (Fig 4). The measured stoichiometry was about 0.9, which confirms the formation of the duplexes in the 1:1 binding ratio. ITC data of Table 2 together with the UV melting data of Table 1 show that the prokaryotic complex is more stable than the eukaryotic one. The prokaryotic complex is characterised by a more negative Δ*H*. The Δ*H* values derived from van’t Hoff analysis of the UV melting profiles (Table 1) and from ITC (Table 2) corroborate with a two-state system. Thermodynamic background of the discrepancies in association enthalpy Δ*H* from both methods is a long-lasting controversy (see e.g. [32, 58–64]). It seems that equality of the enthalpies reflects a simple two-state model of the association (all-or-none) without any coupled equilibria, as long as the temperature dependencies of the enthalpy and entropy terms are taken into account (non-zero Δ*c*_*p*_). On the other hand, discrepancy between these enthalpies suggests that more than two states are involved in the process.

**Table 2:** ITC thermodynamic parameters for the association of the RNA strands forming A-site models.

Model:	Δ*H* [kcal/mol]	TΔ*S* [kcal/mol]	Δ*G* [kcal/mol]	Ka [10^7^ *M*^−1^]	N
prokaryotic	-115.1 (3.7)	-105.1 (4.3)	-10.0 (0.5)	2.4 (1.1)	0.89 (0.02)
eukaryotic	-76.0 (1.8)	-66.5 (2.1)	-9.5 (0.4)	1.1 (0.2)	0.85 (0.02)

Δ*G* and TΔ*S* values calculated for T = 21°C. Values in parentheses represent standard errors from the fitting of the model to the experimental points.

The prokaryotic model is also characterised by a more negative Δ*G*. However, Δ*G* obtained in the UV melting experiments, -18.3 kcal/mol and -14.1 kcal/mol, appear to be overestimated in comparison to the respective -10.0 kcal/mol and -9.5 kcal/mol obtained from the ITC experiments. Note that the Gibbs energy changes are derived directly from the ITC data points, with about 5% uncertainty, while those from the UV measurements are derived indirectly from Δ*H* and *TΔS*, with about 10% uncertainty, due to the propagation of the uncertainties. Nevertheless, the differences of the Δ*G* values are within the range of three standard deviations (3*σ*), and the relative trends of Δ*G*, prokaryotic vs. eukaryotic are similar. In most cases of biomolecular association, Δ*G* weakly depends on temperature, even if both Δ*H* and *TΔS* are strongly temperature dependent, due to enthalpy-entropy compensation, as described e.g., in [65].

### Molecular dynamics simulations of rRNA models

#### Global trajectory measures

**Fig 5:**
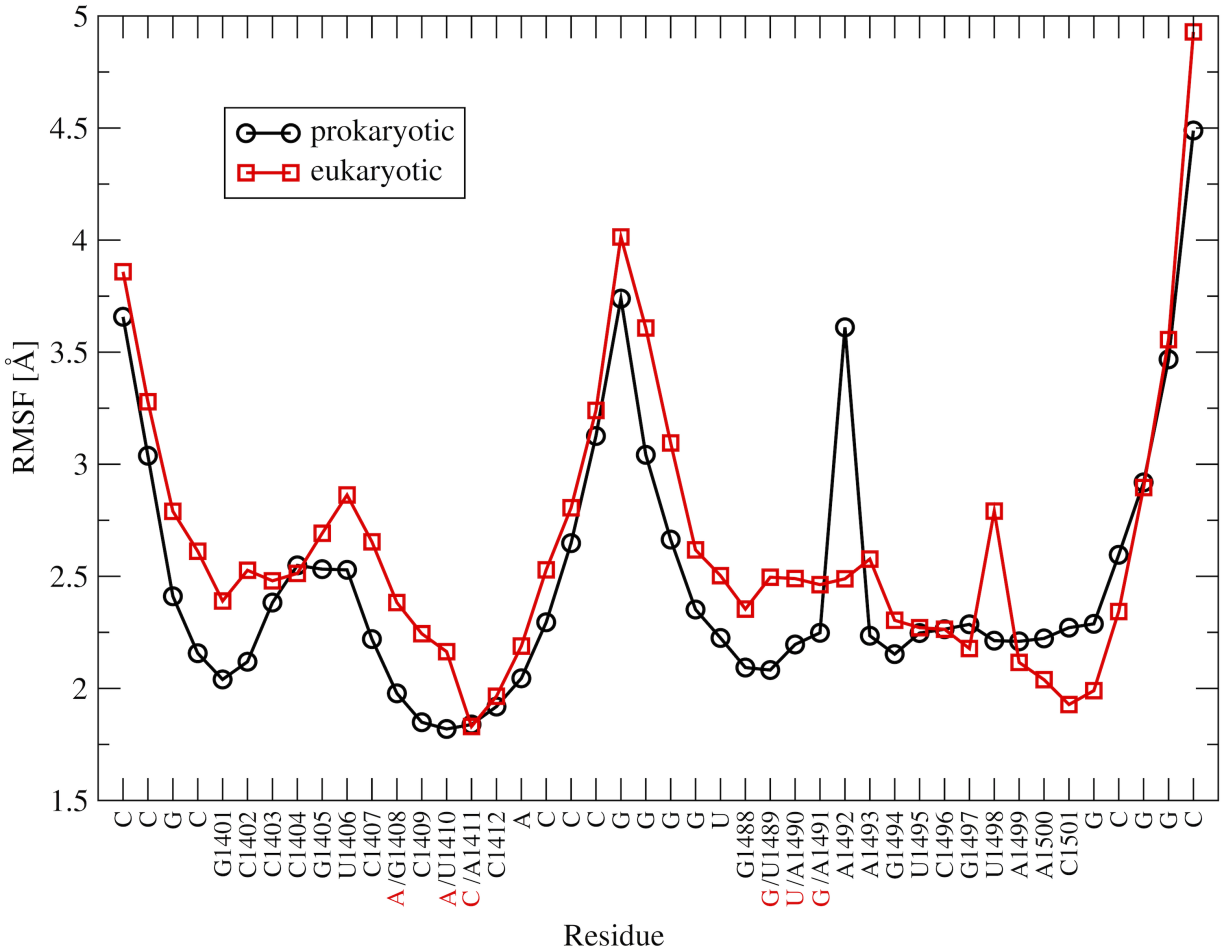
Trajectory derived RMSF for the nucleotides of the prokaryotic and eukaryotic models of rRNA A-site, averaged over 900 ns. For sequences see Fig 2.

In MD simulations both rRNA models preserved overall double-stranded helical conformations in accord with experimental data. Further, the RMSF (root mean square fluctuation) plots of Fig 5 are similar for the prokaryotic and eukaryotic targets, and show increased mobility of the terminal nucleotides. The main difference in RMSF is for nucleotides forming aminoglycoside binding pockets, especially for A1492 (which adopted extra-helical conformation in the prokaryotic and intra-helical in eukaryotic system) and for U1498 (which was intra-helical in prokaryotic and extra-helical in eukaryotic system).

**Fig 6:**
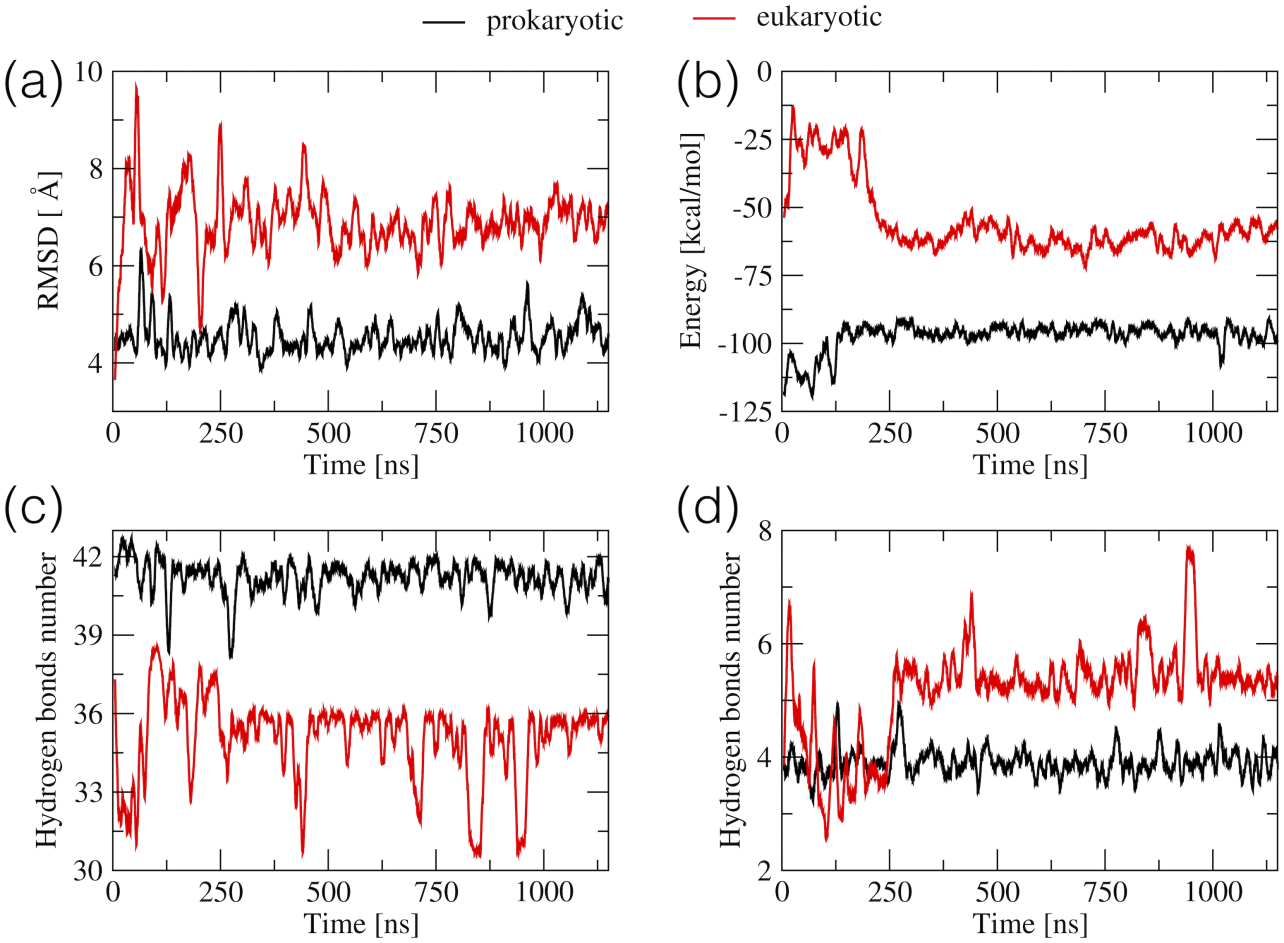
Properties derived from the trajectories of the eukaryotic and prokaryotic RNA models of the decoding A-site. (a) RMSD calculated for RNA heavy atoms with respect to the starting structure, (b) the sum of electrostatic and VdW interactions between the stacking nucleobases, (c) the number of hydrogen bonds in the WC-WC pairs, (d) the number of hydrogen bonds in the non-WC-WC pairs. Each point represents a 5 ns average of each property.

The RMSD plots shown in Fig 6a confirm the overall structural stability of the models. As expected the RMSD obtained for the prokaryotic model, in the 3.8–6.3 Å range, whose initial conformation was from the crystal structure, were lower than for the eukaryotic one, which was obtained by substituting six nucleotides of the prokaryotic model. The number of hydrogen bonds formed by the WC-WC base pairs was higher in the prokaryotic system than in the eukaryotic one (between 38.2 and 42.7 and between 30.5 and 38.5, respectively, Fig 6c). However, for both constructs the numbers fluctuate less after the first 250 ns of the trajectory. The number of non-WC-WC hydrogen bond pairs (Fig 6d) remained the same during the whole simulation of the prokaryotic system, with the average of 3.9. In the eukaryotic system this number decreased from 6.7 to 2.6 in the first 100 ns, for the next 125 ns varied between 2.6 and 4.8, and between 240 and 260 ns increased and remained higher than 5 for the rest of the simulation. This change was due to the intra-helical movement of A1493 described further on.

We have also analysed the sum of stacking interactions detected between all nucleotides (Fig 6b). In terms of the stacking energy both systems stabilize after 250 ns. For the prokaryotic model, stacking stabilizes after 150 ns (with an average of -97 kcal/mol). The eukaryotic system stabilizes between 185 and 250 ns and the stacking energy is in the -51 to -72 kcal/mol range for the rest of the trajectory.

Above measures indicate that in MD simulations, similarly as in the experiments, the eukaryotic system is less stable than the prokaryotic one. The crystal structure of the prokaryotic model adjusts to the *in silico* conditions without any major changes in the base pairing pattern. In the eukaryotic model, in the first 250 ns, we observed some rearrangements and also intra- and extra-helical conformations of bases in the aminoglycoside binding pockets. Therefore, unless stated otherwise, further analyses were performed on the last 900 ns of each trajectory.

**Fig 7:**
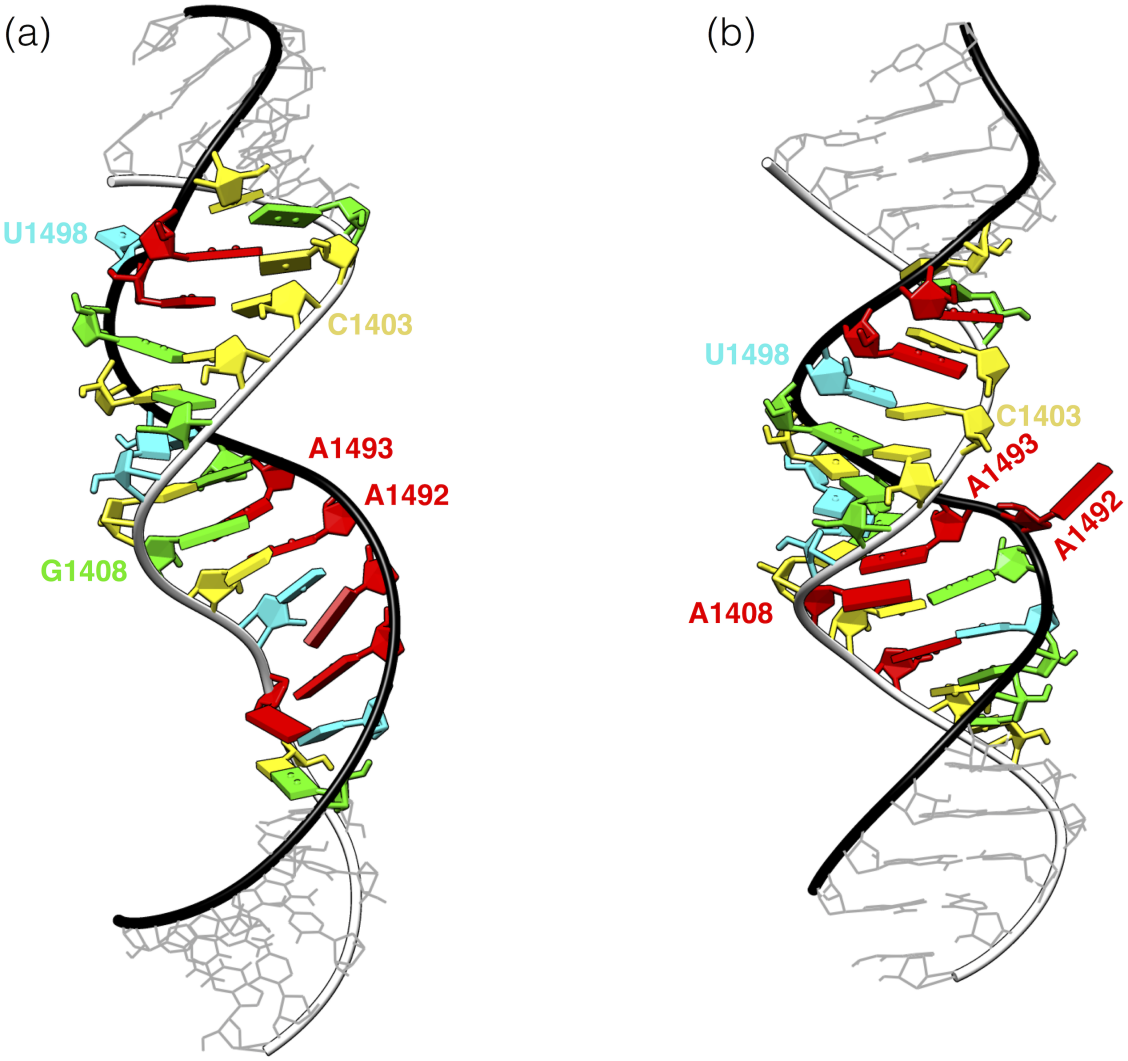
Representative structures of the (a) eukaryotic and (b) prokaryotic RNA models from MD simulations. Central structures from the most occupied clusters with the RMSD criterion of 2 Å. Nucleotides are coloured according to the NDB database convention: A red, U cyan, C yellow, G green [66]. Fragments identical as in the small ribosomal subunits of the relevant organisms a) *H. sapiens*, b) *E. coli* are highlighted. Black ribbon represents the strand complementary to the 2’-O-Me oligonucleotides.

To obtain representative conformations of the systems we performed clustering analysis on 9000 structures from the last 900 ns of each trajectory using the gromos [53] algorithm. Upon applying the same RMSD criterion, the number of clusters roughly corresponds to the conformational variability of the system. As presented in Table 3 the number of clusters increases if the RMSD criterion decreases. Accordingly, regardless of the RMSD criterion, the eukaryotic system has more clusters suggesting it is more structurally variable than the prokaryotic one. The most occupied clusters, calculated with the RMSD criterion of 2 Å, contained 63% and 62% of trajectory frames of the prokaryotic and eukaryotic system, respectively. The central structures of these clusters are shown in Fig 7. Generally, the helix formed in the eukaryotic system is longer and narrower, with a wider major groove than in the prokaryotic one. What is more, representative structures differ in the intra- and extra-helical arrangement of some bases. In the eukaryotic system U1498 remains mainly extra-helical and A1492 intra-helical, while in the prokaryotic system we observed the opposite arrangement.

**Table 3:** The number of clusters determined with different RMSD criteria.

Model:	RMSD = 1.5 [Å]	RMSD = 2 [Å]	RMSD = 2.5 [Å]
prokaryotic	265	40	12
eukaryotic	332	47	16

#### Base pairing pattern in rRNA models

**Fig 8:**
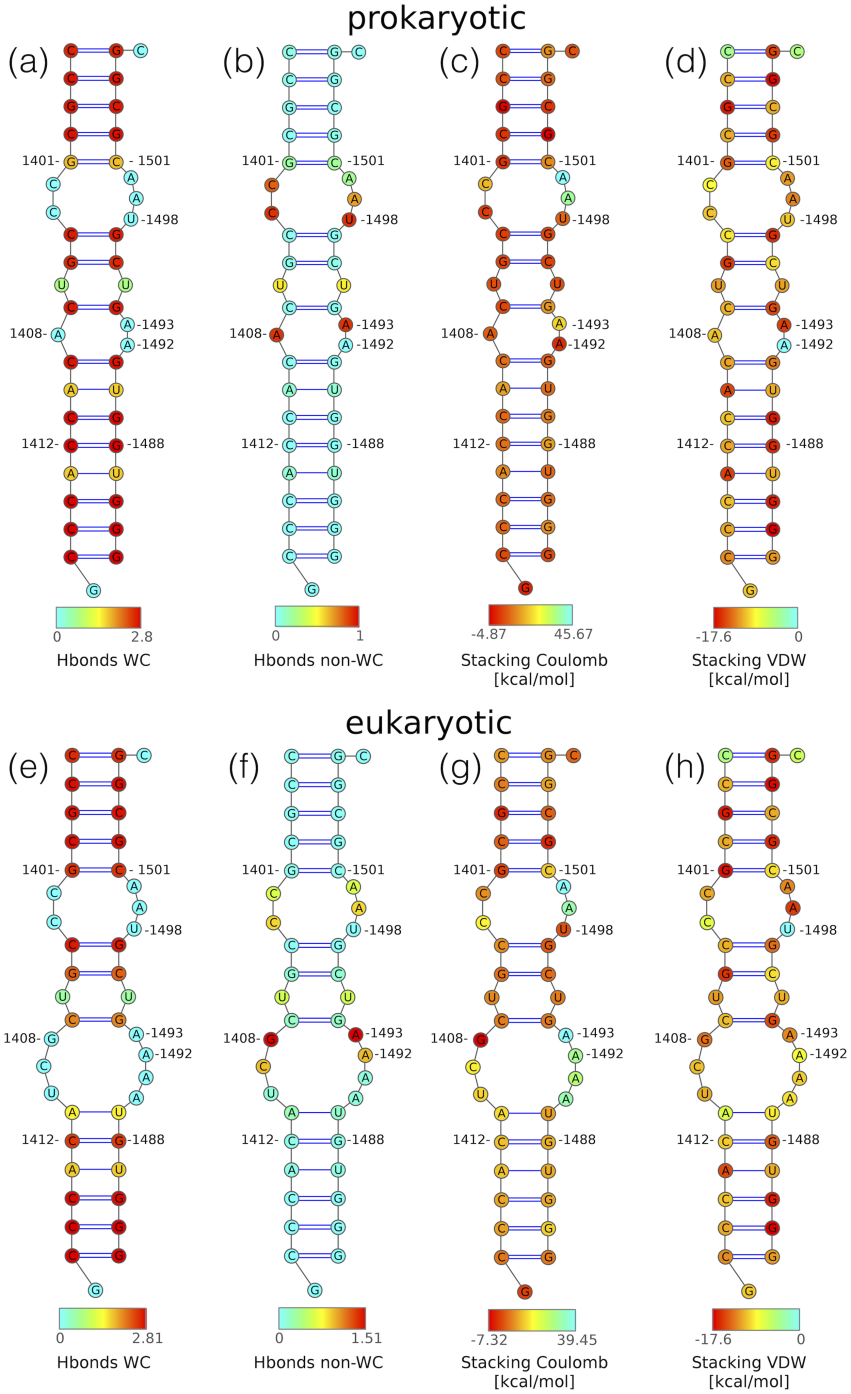
Average secondary structures of the: (a–d) prokaryotic and (e–h) eukaryotic system from the last 900 ns of MD simulations. Only WC-WC pairs that were present in more than 50% of the simulation time are shown. Nucleotides are coloured by the: (a, e) average number of hydrogen bonds in the WC-WC pairs, (b, f) average number of hydrogen bonds in the non-WC-WC pairs, (c, g) average Coulomb energy [kcal/mol], (d, h) average VdW energy [kcal/mol].

Most differences in the WC-WC base pairing were found in the region that differentiates between the models, in between the WC-WC pairs C1407:G1494 and C1412:G1488 (Fig 8a and 8e). In general, in the eukaryotic model, the WC-WC pairs in this region were either broken or less stable than in the prokaryotic system. The mutations that we introduced to obtain the eukaryotic model, resulted in opening of two WC-WC base pairs: C1409:1491G and A1410:1490U (see Fig 8) and enlargement of the bulge in the paromomycin/kanamycin binding region. In the prokaryotic system, the 2-1 asymmetric bulge composed of three adenines: 1408, 1492 and 1493 was present for 96% of the simulation time. In the same region of the eukaryotic model, the larger 4-3 bulge composed of four consecutive adenines 1490-1494, G1408, C1409, and U1410 was present for 63% of simulation time. A detailed comparison of the pairs in the paromomycin/kanamycin binding site in both models is shown in Table 4.

**Table 4:** A list of base pairs in paromomycin/kanamycin binding site. Only pairs observed for more than 5% of the trajectory time and in at least one trajectory are presented. WC, HG and Sugar denote the Watson-Crick, Hoogsteen and sugar edges of the nucleotides involved in the formation of hydrogen bonds. The asterisk indicates that it was impossible to assign the edge uniquely which happens if only one hydrogen bond is formed between the bases and involves the hydrogen positioned at the “corner” of the edges.

Interacting nucleotides:			Pair type	Pair occurrence [% of frames]	
				prokaryotic	eukaryotic
1407	1494	C:G	WC/WC	98%	77%
1407	1494	C:G	WC*Sugar/WC	–	9%
1408	1493	A/G:A	Sugar/WC*HG	53%	40%
1408	1493	A/G:A	Sugar/HG	12%	27%
1408	1493	A/G:A	Sugar/WC	5%	20%
1409	1491	C:G/A	WC/WC	98%	–
1409	1492	C:A	WC*Sugar/WC*HG	–	87%
1410	1490	A/U:U/A	WC/WC	90%	–
1410	1490	A/U:U/A	WC*HG /WC*HG	8%	–
1410	1491	A/U:U/A	WC/Sugar	–	6%

#### Mobility of A1492 and A1493

**Fig 9:**
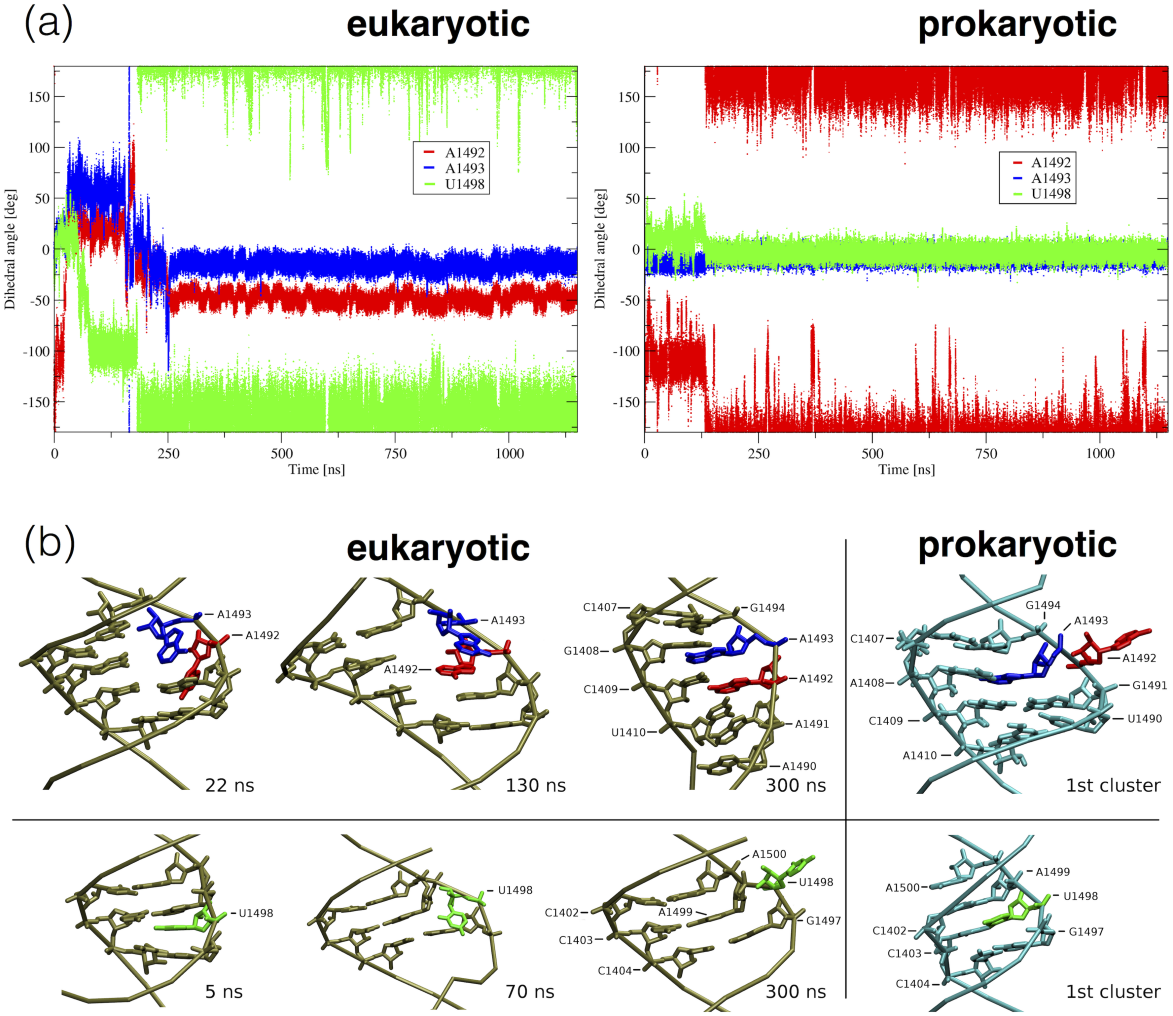
Conformational mobility of A1492, A1493 and U1498. (a) Plots of pseudo-dihedral angles representing the orientation of the nucleobase with respect to the helix backbone for the eukaryotic and prokaryotic model. For angle definition see Methods. (b) Trajectory snapshots presenting the flipping events in the eukaryotic model in comparison with the representative structure of the first most populated cluster from the simulation of the prokaryotic model.

In both systems A1492 and A1493 did not participate in any WC-WC type hydrogen bonds. In the prokaryotic model, A1492 adopted an extra-helical conformation and A1493 an intra-helical one (for the whole simulation time including the first 250 ns, Fig 9). A1492 did not form any hydrogen bond or stacking interactions with other nucleobases. A1493 formed up to three hydrogen bonds with A1408, which were present for 65% of time (for details see Table 4). In addition, A1493 stacked between G1494 and C1409 in entire simulation.

In the eukaryotic construct, A1492 that initially acquired the extra-helical conformation, between 20 and 30 ns of the simulation flipped in through the minor groove (Fig 9). Then, for the next 220 ns both A1492 and A1493 were stacked and moved synchronously. Around 250 ns these adenines found a local minimum and both remained inside the helix till the end of the simulation. A1492 stayed stacked between U1410 and A1493, and A1493 between G1409 and G1494. After the flipping-in, A1492 formed one hydrogen bond with C1409 (the Hoogsteen/Sugar type pair) for 87% of the simulation time. The same type of base pair was formed for 90% of time, between A1493 and G1408, which formed from one to three hydrogen bonds at a time (for details see Table 4).

#### Hygromycin B base pairing pattern

The only WC-WC base pair that was found more stable in the eukaryotic model than in the prokaryotic one is G1401:C1501 (Fig 2). This is also the only WC-WC base pair with affected stability that is not located in or close to the C1407:G1494 or the C1412:G1488 region. This finding corroborates with the differences in the non-WC-WC base pairs in the hygromycin B binding region (Fig 8b and 8f). In the eukaryotic system the G1401:C1501 WC-WC base pair is supported by the neighbouring C1402:A1500 non-WC-WC pair present for 60% of the simulation time. While in the prokaryotic model, C1402 interacts with A1500 only for 21% of time and for another 67% forms the non-WC-WC pair with A1499. The difference is caused mainly by the flipping-out, via the major groove, of U1498 in the eukaryotic system, which occurs between 50 and 180 ns (Fig 9). Thus, C1403 that pairs with U1498 in the prokaryotic system, in the eukaryotic one is bound mainly to A1499. The occurrence of base pairs in the hygromycin B binding pocket is shown in Table 5.

**Table 5:** A list of base pairs in the hygromycin B binding site. Only pairs occurring more than 5% of the simulation time and in at least one trajectory are listed. WC, HG and Sugar denote, respectively, the Watson-Crick, Hoogsteen and sugar edges of the nucleotides involved in the formation of hydrogen bonds. The asterisk means that it was impossible to assign the edge uniquely which happens if only one hydrogen bond is formed between the bases and involves the hydrogen positioned at the “corner” of the edges.

Interacting nucleotides:			Pair type	Pair occurrence [% of frames]	
				prokaryotic	eukaryotic
1402	1499	C:A	WC*Sugar/WC*HG	67%	–
1402	1500	C:A	WC*Sugar/WC*HG	21%	55%
1403	1498	C:U	WC*HG /WC*Sugar	90%	–
1403	1499	C:A	WC*Sugar/WC*HG	–	76%
1404	1497	C:G	WC/WC	98%	98%
1405	1496	G:C	WC/WC	96%	85%
1405	1496	G:C	WC*Sugar/WC*Sugar	2%	6%
1406	1495	U:U	WC*HG /WC	43%	49%
1406	1495	U:U	WC/WC	35%	25%
1406	1495	U:U	WC/WC*HG	6%	1%
1406	1495	U:U	WC/WC*Sugar	4%	6%

#### Stacking interactions

Stacking interactions between nucleotides were calculated taking into account both the electrostatic and van der Waals energy terms of the stacked nucleobases. Overall, as presented in Fig 6b, the nucleotides in the eukaryotic system are less stacked than in the prokaryotic one, mainly due to the less favourable electrostatic interactions caused by the presence of four consecutive adenine bases in the eukaryotic model. The major difference in stacking is in the paromomycin/kanamycin and hygromycin B binding site bulges. This is best illustrated for the 1490 and 1493 region, where each of the consecutive adenines in the eukaryotic system has by about 20 kcal/mol higher electrostatic energy than the corresponding base in the prokaryotic model (Fig 8c and 8g). The most interesting reverse case is the G1408A mutation; in the eukaryotic system G1408 has more favourable electrostatic interactions than the corresponding A1408 in the prokaryotic system.

Considering only the vdW energy contributions of the stacked bases, both systems were similarly stacked. The sum of the vdW interaction energy term averaged over 900 ns is -271 ± 6 kcal/mol for the prokaryotic and -263 ± 7 kcal/mol for the eukaryotic system. The largest differences were found for the nucleotides that adopted an intra-helical conformation in one system and extra-helical in another system, namely A1492 and U1498 (Fig 8d and 8h). In both systems the most favourable stacking was found in regions with the set of purines present on opposite strands at a distance of one helical step (e. g., see G1401, G1502, G1399, G1504 and G1486, A1413, G1488 in both systems in Fig 8c, 8g, 8d and 8h).

#### Stabilities of the regions targeted by the 2’-O-methylated oligomers

Considering the number of hydrogen bonds, the most stable prokaryotic fragment is the one targeted by oligonucleotide 1489 (Fig 2). The average number of hydrogen bonds formed per nucleotide in this region is 1.83. The rRNA targets of 1490 and 1491 oligomers form on average 1.62 and 1.47 hydrogen bonds per nucleotide, respectively. The same ordering of targets was obtained considering the nucleotide-averaged stacking energies (see Table 6). The most stacked target is the one for the 1489 oligonucleotide.

**Table 6:** Average stacking interactions per nucleotide in the regions targeted by 2’-O-methylated oligomers in prokaryotic and eukaryotic decoding A-site models.

Model:	Oligomer target:	Coulomb [kcal/mol]	VdW [kcal/mol]	Total [kcal/mol]
prokaryotic	1489	7.65 (1.36)	-12.08 (0.43)	-4.44 (1.34)
	1490	10.38 (1.65)	-11.69 (0.43)	-1.31 (1.59)
	1491	14.14 (1.75)	-11.74 (0.44)	2.39 (1.71)
eukaryotic	1489	14.62 (1.67)	-10.09 (0.51)	4.53 (1.65)
	1490	17.05 (1.74)	-10.71 (0.52)	6.34 (1.71)
	1491	17.77 (1.63)	-11.03 (0.49)	6.75 (1.63)

Values (with standard deviation in parenthesis) derived from MD simulations.

For the eukaryotic target, the order of target stability depends on the assessed property. Hydrogen bonds suggest that the most stable eukaryotic fragment is the one aimed as target for the 1489 oligomer, forming on average 1.27 bonds per nucleotide. The next are the 1491 and 1490 targets, with 1.24 and 1.19 bonds per nucleotide, respectively. Considering the average stacking energy, the order is the same as in the prokaryotic model, namely, 1489, 1490 and 1491 (Table 6).

Note that in some cases the total stacking interaction presented in Table 6 is positive. This happens because the stacking energy was assumed as a sum of the force field electrostatic and VdW energy terms between atoms of geometrically overlapping nucleobases. Some additional terms, such as solvation energy are omitted, so the values of Total stacking in Table 6 may be positive even for a stable conformation of nucleotides because the positive term origins only from the electrostatic term. This term is based on a single-point partial charge model of the force field and may be either positive or negative depending on the orientation of nucleobases. This means that even small changes in the positioning of bases during simulations result in large changes in electrostatic contribution to stacking, which is visible in the large standard deviations from the average. However, histograms of results of Table 6 that are shown in S2 Fig confirm that the averages are meaningful.

### Solution studies of the interactions of 2’-O-methylated oligomers with RNA targets

We further investigated the interactions of the 2’-O-Me RNA oligomers with the decoding A-site rRNA models experimentally to find which oligonucleotide forms the most stable complex with the prokaryotic model and at the same time does not bind to the eukaryotic model. By design, all three oligonucleotides are fully complementary to the prokaryotic A-site and possess one, two or three mismatches toward the eukaryotic one (Fig 2). We used fluorescence spectroscopy and gel electrophoresis to confirm the binding of these oligonucleotides to the targets, as well as ITC and UV-monitored melting to estimate the thermodynamic stability of the complexes. We monitored the oligonucleotide interactions both with double-stranded and with single-stranded rRNA fragments.

#### Double-stranded decoding A-site rRNA models

**Fig 10:**
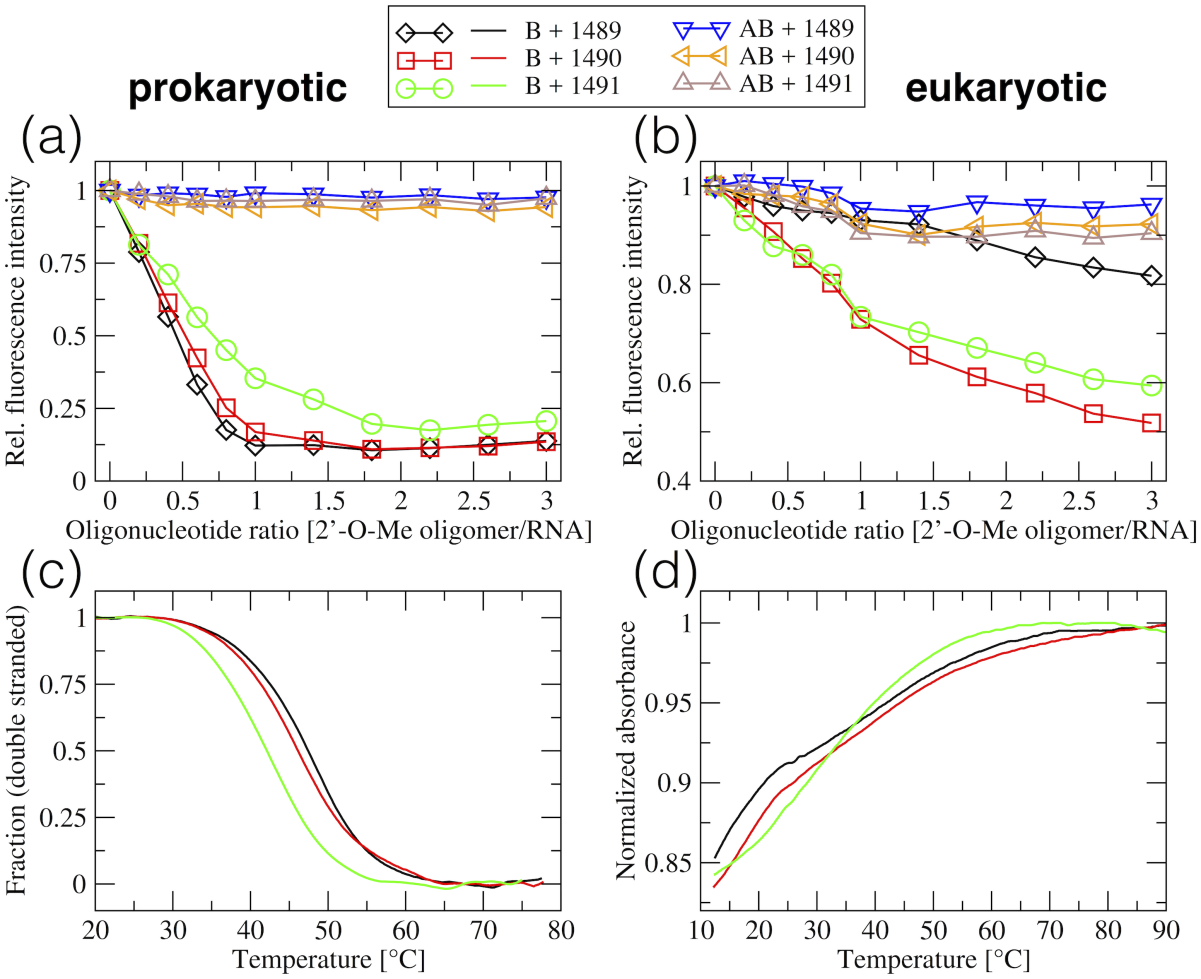
Solution studies of the interactions between the 2’-O-Me oligomers and rRNA targets. (a, b) Fluorescence of 2AP1493 in the rRNA strand B monitored during titration of 2’-O-Me oligomers to the rRNA targets. B stands for the single B strand of the rRNA model, AB stands for the double stranded model. (c, d) UV melting profiles of double-stranded models (consisting of rRNA strand B and 2’-O-Me oligomers) showing the fraction of the double strands as a function of temperature (c) and normalized absorbance (d) since fitting was impossible due to the lack of plateaus. For the sequences and names refer to Fig 2.

We did not detect binding of 2’-O-Me oligomers to double-stranded models of the prokaryotic and eukaryotic decoding A-sites. PAGE experiments show that in the samples containing the double-stranded rRNA and 2’-O-Me oligomer (S1 Fig, lane 2), we did not observe any band migrating slower than the double-stranded rRNA target, what would indicate a triplex. Furthermore, in these samples, the band representing the unbound 2’-O-Me oligomer was always present. There was also no indication of the formation of triplexes in the fluorescence experiments. Adding 2’-O-Me oligomers to the double-stranded targets (Fig 10a and 10b) changed the fluorescence of 2AP1493 only slightly. The fluorescence signal drops are significantly lower than those observed if oligomers bind to single-stranded rRNA (see below).

#### Formation of the duplexes involving 2’-O-Me oligomers

**Fig 11:**
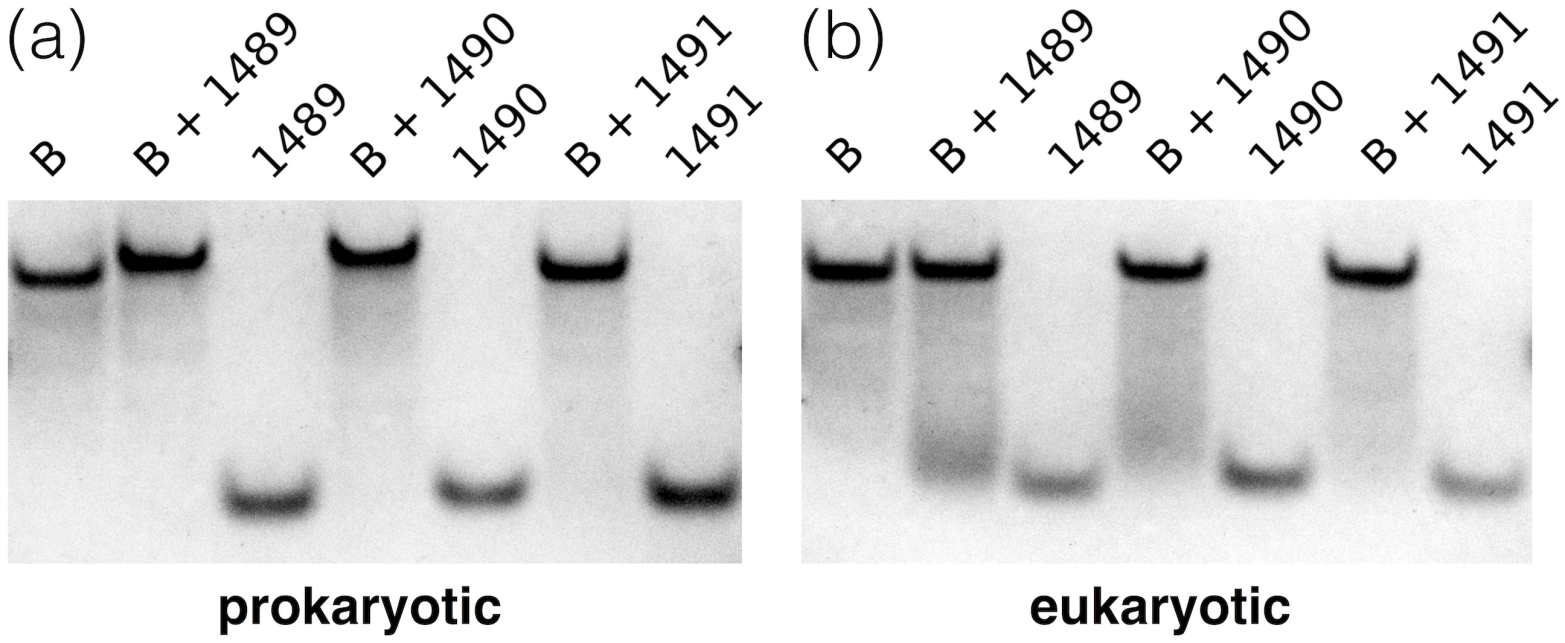
PAGE studies of the interactions between the 2’-O-Me oligomers and strand B of the (a) prokaryotic and (b) eukaryotic rRNA targets. For the sequence of strand B and oligomers see Fig 2.

We verified the binding of 2’-O-Me oligomers to both A and B strands of the prokaryotic and eukaryotic sequences of the A-site models. The PAGE experiments confirmed that oligomers did not form complexes with non-complementary A strands (see S1 Fig) but all three 2’-O-Me oligomers fully hybridized with the complementary B strand of the prokaryotic model (Fig 11a). In all samples containing both the rRNA strand B and complementary 2’-O-Me oligomer, there was no band from the oligomer. Moreover, for the 1489 and 1490 oligomers that were mixed with the B strand, the only visible band migrated slightly slower than strand B alone.

However, the gels obtained for 2’-O-Me oligomers and strand B of the eukaryotic target are not clear (Fig 11b). Samples containing both the rRNA and 2’-O-Me oligomers did not migrate slower but similarly as the strand B alone. In addition, in the sample with the B strand and oligomer 1489 (the one with three mismatches against the eukaryotic target), the band from the oligomer was present. This suggests that not all oligomers fully hybridized with the partially complementary B strand of the eukaryotic rRNA target.

The fluorescence studies monitoring the interactions of the 2AP-labeled B strand with the 2’-O-Me oligomers (Fig 10a and 10b), confirm PAGE results. Full hybridization of all three 2’-O-Me oligomers with the B strand of the prokaryotic rRNA model was indicated by a steady decrease of the 2AP1493 fluorescence intensity up to the 1:1 strand ratio (Fig 10a). Interactions with the B strand of the eukaryotic rRNA model were detected only for oligonucleotides 1490 and 1491 (Fig 10b) but were weaker. For these oligomers there is an inflection of the fluorescence curve at the 1:1 ratio. However, after reaching the 1:1 ratio, the 2AP fluorescence signal does not reach equilibrium as in the complexes with the prokaryotic target. The curve for the 1489 oligomer is similar to the curves observed while targeting the double-stranded rRNA, which together with the PAGE results, suggests that oligomer 1489 does not form a complex with the B strand of the eukaryotic rRNA model.

#### Thermal stability of double-stranded complexes

The stability of the complexes formed by the 2’-O-Me RNA oligomers and strand B of the rRNA models was examined by thermal melting and ITC. In the UV-monitored melting experiments all duplexes involving the prokaryotic rRNA showed biphasic transitions. Fitted sigmoidal functions of the fraction of double-stranded structures versus temperature are shown in Fig 10c and T_*m*_ and thermodynamic parameters in Table 7. The most thermally stable complex was created by the oligomer 1489 and the least stable by 1491.

**Table 7:** Thermodynamic parameters for the duplexes formed by 2’-O-Me RNA oligomers with strand B of the prokaryotic rRNA target, derived from the UV melting profiles.

Oligomer:	Δ*H* [kcal/mol]	TΔ*S* [kcal/mol]	Δ*G* [kcal/mol]	*T*_*m*_ [°C]
1489	-72.9 (0.9)	-58.5 (0.6)	-14.4 (1.5)	47.4 (0.5)
1490	-68.7 (0.7)	-54.8 (0.6)	-13.9 (1.3)	46.1 (0.5)
1491	-73.0 (0.8)	-59.7 (0.6)	-13.3 (1.4)	42.1 (0.5)

Δ*G* and TΔ*S* values calculated for T = 21°C. Values in parentheses represent standard errors from three experiments.

UV-melting curves with the oligomers and B strand of the eukaryotic rRNA model did not show a biphasic transition and low temperature plateaus (Fig 10d). Thus, we could not fit the two-state model with slopping baselines to these data and calculate the melting temperatures or thermodynamic parameters. Therefore, we present normalised absorbance versus temperature (Fig 10d); from these curves we estimated the melting temperatures to be lower than 33°C.

**Fig 12:**
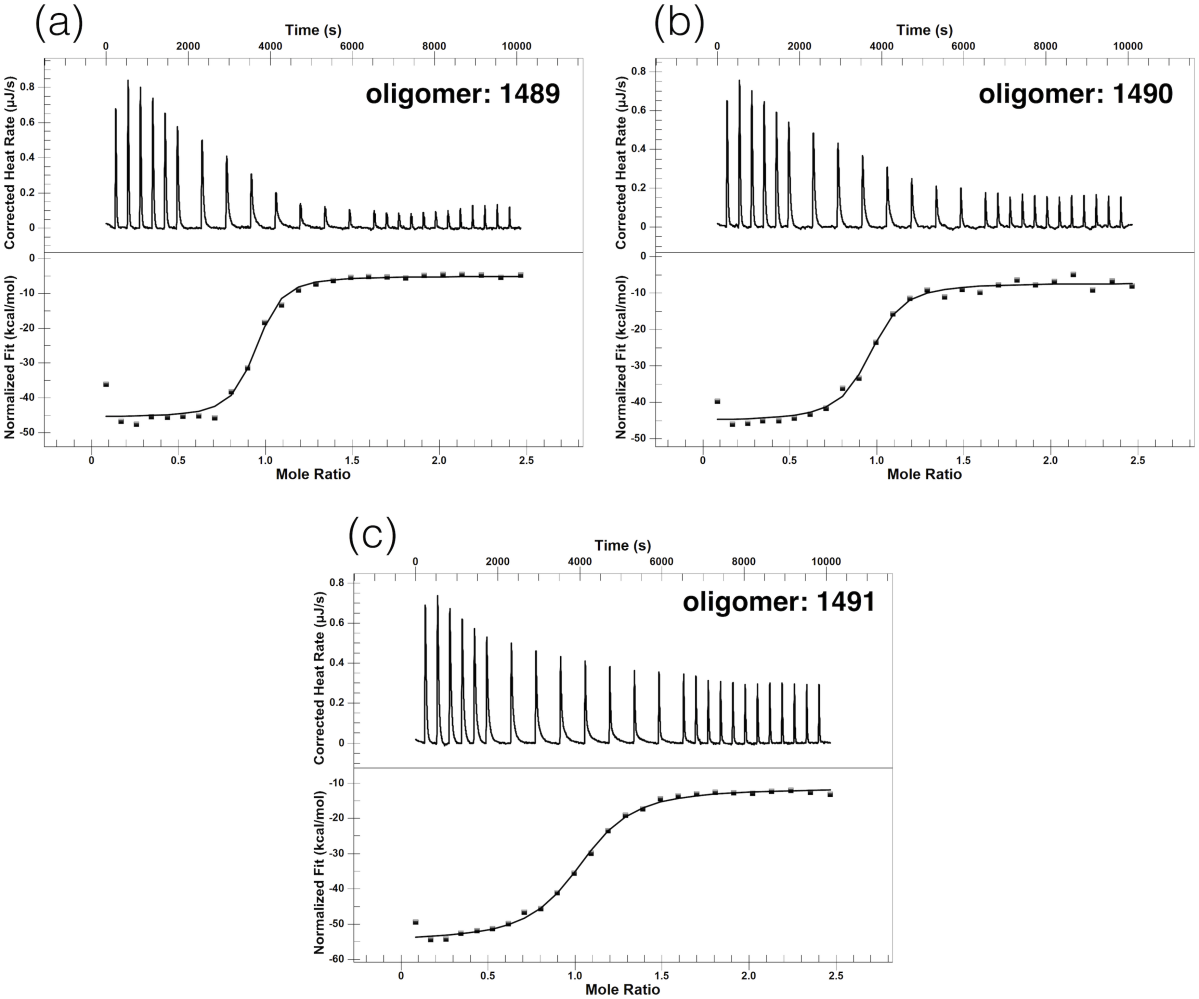
ITC binding scans showing the titration of 2’-O-Me-RNA oligomers to strand B of the prokaryotic rRNA model at T = 21°C. (a) oligomer 1489, (b) oligomer 1490, (c) oligomer 1491.

With ITC we investigated the stabilities of the complexes formed by the oligomers with prokaryotic strand B (Fig 12). The times needed for the systems to equilibrate after injections were noticeably longer than in the experiments in which the B strands were titrated to the A strands (Fig 4). That is why the time between the injections was set between 5 to 10 minutes to ensure equilibration after each injection. The longest time was used in the area where the binding curves were expected to transition from the lower to upper plateau. For all oligomers the obtained reaction stoichiometries were close to one, which together with the fluorescence experiments (Fig 10c) confirms the 1:1 binding ratio. Thermodynamic parameters obtained from ITC data (Table 8) show the same order of Δ*G*s as derived from the UV melting experiments (Table 7). In both methods the most stable complex considering *T*_*m*_ and Δ*G*s was formed by oligomer 1489 and the least stable one by 1491.

**Table 8:** ITC thermodynamic parameters for the duplexes formed by oligomers with strand B of the prokaryotic rRNA model.

Oligomer:	Δ*H* [kcal/mol]	TΔ*S* [kcal/mol]	Δ*G* [kcal/mol]	Ka [10^7^ *M*^−1^]	N
1489	-40.6 (2.5)	-30.8 (1.5)	-9.8 (0.5)	1.9 (1.5)	0.91 (0.02)
1490	-37.8 (2.1)	-28.3 (1.4)	-9.5 (0.5)	1.2 (0.7)	0.93 (0.02)
1491	-43.2 (1.1)	-34.3 (1.5)	-8.9 (0.4)	0.5 (0.1)	1.01 (0.02)

Δ*G* and TΔ*S* values calculated for T = 21°C. Values in parentheses represent standard errors from the fitting of the model to experimental points.

However, Δ*H* and Δ*S*, derived from the analysis of UV melting curves, are about twice as large as from ITC experiments, contrary to thermodynamic parameters of the RNA strands (see Tables 1 and 2). This points to a more complex model of strand association than a two-state model and possible presence of some intermediate forms of the complexes.

For Δ*G*s the ratio between UV and ITC experiments is about 1.5. The differences of Δ*G* values are within the range of three standard deviations (3*σ*), and the relative trends of Δ*G* for the three 2’-O-Me RNA oligomers are preserved, as for the A-site rRNA models (see above).

## Discussion

### Thermal stability and hydrogen bond pattern in the helix 44 target

#### Molecular dynamics simulations analysis

A common practice is to perform three or more shorter MD simulations of the same system and then consider them as independent measurements. This approach provides better sampling, estimates of convergence, and uncertainties of reported measures. Here we report only one simulation per system but trajectories are sufficiently longer than reported in previous studies of similar RNA systems [28, 29, 67–69]. Nevertheless, we used the average block analysis method [70, 71] to test if the simulations were long enough to observe that if each trajectory is divided into blocks, the blocks are independent of one another. The plots of Blocked Standard Error (BSE) versus block length for the RMSD (Fig 6a) and sum of VdW and electrostatic interaction between stacked nucleobases (Fig 6b) are presented in S3 Fig). The shapes of curves, BSE increases monotonically and asymptotes to the value of standard deviation of analyzed quantity, show that the blocks are statistically independent (block length is substantially greater than the correlation time). This suggest that presented simulations can be considered as converged [72].

While no reliable single measure is known that would prove that a simulation has converged [73], we suggest to consider parameters other than RMSD to estimate the time that an RNA system requires for relaxation from the starting structure. For example, in both simulations presented in this study, the analysis of stacking versus time curves revealed conformational transitions related to the nucleotide rearrangement from the starting structure, which were not visible in the RMSD curves (see Fig 6).

#### Overall thermal stability of RNA targets

In both experiments and simulations we observed that the eukaryotic helix 44 fragment is less thermally stable than the prokaryotic one. This observation corroborates with the study that compared the *in situ* accessibility of the small subunit rRNA to DNA oligonucleotide probes among bacteria, archea and eukarya domains. This helix 44 rRNA fragment was found more accessible to DNA probes in eukaryotic cells than in prokaryotic ones [74].

The overall lower stability of the eukaryotic-like construct may be explained by a different base pairing pattern, mainly the lack of two G:C base pairs that are present in the prokaryotic model (Fig 2). In the eukaryotic system the C1409:G1491 base pair closing the bulge is replaced by C1409:A1491 and the C1411:G1489 pair in the stem by A1411:U1489. Our previous MD studies of smaller rRNA constructs, containing only the decoding A-site without the hygromycin B binding pocket, have shown that even lack of one canonical C1409:G1491 pair in the bulge enables larger tertiary structural freedom and destabilisation of the complex of the human decoding A-site variant [29].

In general, the presented model of the eukaryotic, human like, fragment of helix 44 in the small ribosomal subunit, is a stable construct that can be used to monitor ligand binding to the eukaryotic 16S RNA decoding site. Furthermore, this is the eukaryotic A-site model containing two bulges: one accommodating 2-DOS aminoglycosides, such as neomycin, paromomycin, kanamycin or gentamicin and the other one accommodating hygromycin B. The constructs containing two bulges may become helpful in studying aminoglycoside modifications that extend beyond their primary binding site.

#### Decoding A-site adenines in the prokaryotic helix 44 model

In our MD simulations A1492 acquired extra-helical conformations and A1493 was flipped in. This extra-helical conformation of A1492, present in the starting crystal structure (PDB code: 3LOA), was described as a result of the involvement of A1492 in crystal packing contacts, which may bear little relevance to its conformational states in solution [6]. This result was distinct from the arrangements observed previously in the structures of the whole 30S ribosomal subunit [75, 76], in which both adenines were found in the intra-helical conformations. Our previous MD studies have also shown that A1492 prefers intra-helical states [28, 68, 69]. However, an identical conformation of these adenines, as in our MD trajectory, was observed previously in other decoding A-site crystallographic models: (i) in the empty site of dimeric A-site constructs (PDB code: 2ET8) which were complexed with aminoglycoside ligands at only one of the two decoding sites [77, 78] and (ii) in one site of the ligand-free dimeric decoding A-site construct (PDB code: 3BNL) [79]. What is more, in the latter construct, the unusual conformation of these adenines (A1492 flipped-out and A1493 flipped-in) was preserved in 2 out of 12 MD simulations for a simulation time between 150 and 300 ns [29]. Interestingly, in a 30 ns MD study of a longer part of helix 44 than mentioned above, in which both A1492 and A1493 were in the intra-helical conformations in the starting structure, two A1492 flipping-out events in 17 ns were observed [67]. Thus it seems that the positioning of these adenines in ligand-free systems is sensitive to both simulation and experimental conditions and confirms the required conformational freedom of this decoding A site switch.

However, the persistence of A1492 outside the helix is unexpected since the flipped-in conformations of both A1492 and A1493 were shown to be slightly energetically preferred [2, 3] and the free energy barrier for flipping out was slightly lower for A1493 than for A1492. Interestingly, the range of pseudo-dihedral angles sampled by these adenines in the prokaryotic simulation (Fig 9) overlaps with the local minimum of their free energy landscape along pseudo-dihedral angles obtained for ligand free bacterial A-site model in the umbrella sampling MD simulations [2]. Thus, preservation of the extra-helical state of A1492 in the simulation of the prokaryotic model may be due to trapping in the local minimum. While this was caused by the initial configuration, it suggests sampling limitations of MD simulations or overestimated stability of RNA molecules in the used force field.

#### Decoding A-site adenines in the eukaryotic helix 44 model

In the eukaryotic model, A1492, which was initially in the extra-helical conformation, flipped in through the minor groove and remained inside the helix till the end of the simulation. A1493 preserved the intra-helical state in the whole simulation, forming a stable Sugar/Hoogsteen base pair with G1408. A similar behaviour of these adenines, flipping-in of A1492 and forming the A1493:G1408 base pair, was recently reported in unrestrained simulations of a smaller model of the human decoding A-site [29].

Despite the formation of long lasting hydrogen bonds by A1492 and A1493 in the eukaryotic decoding A-site bulge, they were less stacked than in the prokaryotic system. This may be due to the unfavourable electrostatic interactions caused mainly by the sequence of four consecutive adenines A1490–A1493. Unfavourable stacking interactions of these adenines in the eukaryotic complex follow from the measured higher fluorescence intensity of the 2AP-labeled A1493 during the formation of a double stranded complex (as compared with the prokaryotic system, Fig 3). Similar difference in the fluorescence intensity, between the free prokaryotic and eukaryotic decoding A-site models was observed also for the 2AP-labeled A1492 [80]. This is in agreement with the recent observation of repetitive destacking events of A1492 seen in the MD study of the smaller model of the eukaryotic A-site [29].

#### Dynamics of hygromycin B binding site

**Fig 13:**
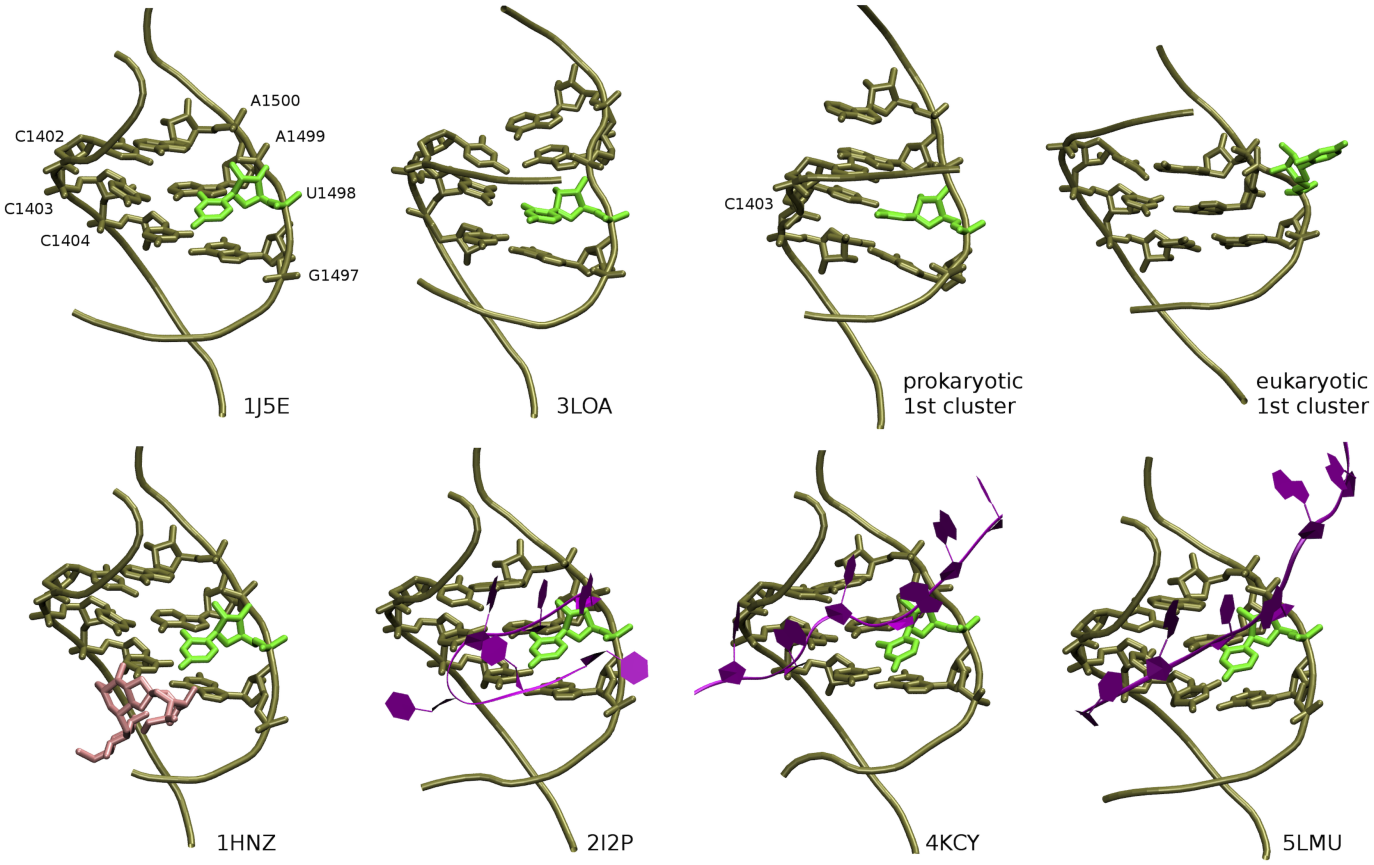
The C1402:C4104 and G1497:A1500 region of hygromycin B binding site in the crystal and cryo-electron microscopy structures of the prokaryotic small ribosomal subunit. U1498 is in light green, hygromycin B in pink and mRNA in violet. PDB codes are given next to each structure. Prokaryotic and eukaryotic “1st cluster” refers to representative structures of the most occupied clusters obtained in MD simulations with the RMSD criterion of 2 Å.

Besides A1492 and A1493, the dynamics of hygromycin B binding site was also different in the studied models. Different base pairing pattern was caused mainly by the flip-out of U1498 in the eukaryotic system. This U1498 flip-out occurred through the major groove between 50 and 180 ns of the trajectory. In the prokaryotic model, U1498 was in the intra-helical conformation for the whole simulation, interacting with C1403 for most of the time (Fig 13 prokaryotic 1st cluster image). Since the conformation of hygromycin B binding pocket in the 3LOA crystal structure (used by us as a starting point for MD simulations) was found nearly identical as in the ribosome subunit structures [6] (both with [13] and without hygromycin B [75]), we did not expect flipping out of this uracil. However, our comparison of the C1402:C1404 to G1497:A1500 region in the 3LOA structure with the same region in the structures of ribosome subunits (Fig 13) revealed a distinct base-pairing pattern.

The C1403:U1498 pair, observed in the 3LOA structure, was not found in the following crystal structures of the small ribosomal subunit: with (1HNZ) and without (1J5E) hygromycin B and with mRNA in various stages of translation (2I2P, 2I2U [14] and 4KCY, 4KBT, 4KD8, 4KDG [15]), as well as in the recent cryo-electron microscopy-derived structures (5LMN, 5LMO, 5LMP, 5LMQ, 5LMR, 5LMS, 5LMT, 5LMU, 5LMV [81]). What is more, in all these structures, U1498 faces towards a major groove as in the initial stage of the flip-out in our MD simulation of the eukaryotic model (see Fig 9), which in some cases leads to the interaction of U1498 with mRNA. While the conformation observed in the eukaryotic model appears to better reproduce the ribosomal base-pairing pattern, hygromycin B binding pockets in both models may be affected by the G:C basepairs, above the G1401:C1501 pair that are not present in the ribosome context (Fig 2).

### Interactions of 2’-O-Methylated oligonucleotides with the rRNA targets

#### The structures of complexes

The rRNA models used here, due to the additional terminal G:C base pairs (Fig 2), may be less susceptible to strand invasion than the corresponding rRNA sequence in the ribosome context. In the structures of the 30S bacterial subunit, the G1401:C1501 base pair is the second before last base pair in helix h44. On the other side, the C1412:G1488 pair in the ribosome structures is followed mainly by non-canonical pairs, in contrast to the stable G:C pairs in the models that we used.

At the same time, the enhanced stability of the duplexes, makes these models ideal targets to form a triple-stranded helix via Hoogsteen interactions, because this binding mode does not require unwinding of the targeted duplex RNA. However, we did not observe any binding of 2’-O-Me oligomers to the eukaryotic and prokaryotic A-site models, which suggests that the formation of a triplex via Hoogsteen base-pairing, is less probable. This agrees with the fact, that triplex formation preferentially requires a polypurine-polypyrimidine sequence in the targeted duplex [82], which is not present in our systems. Also, it was found that incorporation of 2’-O-Me nucleotides into a triplex-forming oligonucleotide destabilizes its binding to an RNA duplex region [83]. With this in mind, we conclude that the studied 2’-O-methylated RNA oligomers bind to the rRNA via strand invasion. This conclusion is supported by the fact that all oligomers bound tightly to the complementary B strand of the prokaryotic rRNA A-site model forming stable 1:1 duplexes. Nevertheless, because the studies on thermodynamics and kinetics of RNA strand displacement are less advanced than for DNA [84], the detailed structure of the complex being formed in the ribosome context still needs elucidation.

#### Target selectivity

All studied 2’-O-Me oligomers are fully complementary to the bacterial rRNA, but posses one, two or three mismatches toward the eukaryotic target. In the UV melting experiments, the oligomers did not form a stable complex with the complementary strand of the eukaryotic target. However, only one oligomer, 1489, with three mismatches, did not show any interaction with the eukaryotic target in the fluorescence and PAGE experiments. Interestingly, the 1489 oligomer, apart from having the best target selectivity, has also shown the best affinity to the single-stranded prokaryotic rRNA, which makes it the best candidate for further studies. However, MD analysis of both targets shows that the rRNA sequence targeted by this oligomer is the most stable one, which may lower the strand invasion abilities. While our experiments were performed under low salt concentrations, which do not enhance the formation of the complexes between nucleic acid oligomers, we conclude that the 2’-O-Me oligomers targeting functional sites of bacterial rRNA, should possess at least three mismatches to the corresponding sequence in eukaryotic ribosomes.

## Conclusions

We examined the dynamics of the prokaryotic and eukaryotic models of 16S rRNA helix 44 containing two 2-DOS aminoglycoside binding sites (paromomycin/kanamycin and hygromycin B) and the interactions of these models with 2’-O-Me RNA oligomers. The eukaryotic model was built by introducing six mutations to the prokaryotic one. Both experiments and MD simulations have shown that the eukaryotic model is overall less conformationally stable (in terms of hydrogen bonding and stacking interactions) than the equivalent prokaryotic model. We have also characterised for the first time the dynamics of hygromycin B binding site in the models of both prokaryotic and eukaryotic rRNA showing differences in flexibility of U1498 between the models.

In solution studies performed on 2’-O-Me oligomers and rRNA models of the prokaryotic and eukaryotic aminoglycoside binding sites, we did not observe binding of 2’-O-me RNAs to double stranded rRNAs. However, the 2’-O-Me oligomers interacted strongly with complementary single strands. Experiments suggest that 2’-O-Me RNAs bind to rRNA via strand invasion and that for targeting functional sites of bacterial rRNA with 2’-O-Me oligoribonucleotides at least three mismatches are necessary to assure for appropriate target selectivity. The 1489 oligomer had the best affinity toward the prokaryotic RNA strand and did not specifically interact with eukaryotic target making it a good candidate for further modifications.

## Supporting information

S1 Fig PAGE studies of 2’-O-Me oligoribonucleotides targeting prokaryotic and eukaryotic rRNA A-site models. For the rRNA and 2’-O-Me RNA sequences see Fig 2 in the main text.
(TIFF)

S2 Fig Histograms of average stacking interactions per nucleotide in the regions targeted by 2’-O-Me oligomers in prokaryotic and eukaryotic decoding A-site models. For average and standard deviation values see Table 6 in the main text.
(TIFF)

S3 Fig Standard error estimate for the RMSD and sum of VdW and electrostatic interaction between stacked nucleobases obtained with the average block analysis method. a) RMSD of the prokaryotic model, b) RMSD of the eukaryotic model, c) stacking energy of the prokaryotic model, d) stacking energy of the eukaryotic model. For the time-series of these values see Fig 6a) and 6b) in the main text. The Block Standard Error (BSE) values are plotted as a function of the block size (black line). In addition, the analytical block average curves (red line) are plotted with the assumption that the autocorrelation is a sum of two exponentials (see [71] in the main text for details and complete derivation).
(TIF)

## References

[1] Demeshkina N, Jenner L, Westhof E, Yusupov M, Yusupova G. A new understanding of the decoding principle on the ribosome. Nature. 2012;484(7393):256–259. doi: 10.1038/nature10913 22437501 10.1038/nature10913

[2] Zeng X, Chugh J, Casiano-Negroni A, Al-Hashimi HM, Brooks CL. Flipping of the ribosomal A-site adenines provides a basis for tRNA selection. J Mol Biol. 2014;426(19):3201–3213. doi: 10.1016/j.jmb.2014.04.029 24813122 10.1016/j.jmb.2014.04.029 PMC4150856

[3] Sanbonmatsu KY. Energy landscape of the ribosomal decoding center. Biochimie. 2006;88(8):1053–9. doi: 10.1016/j.biochi.2006.06.012 16905237 10.1016/j.biochi.2006.06.012

[4] Zhang W, Dunkle JA, Cate JHD. Structures of the ribosome in intermediate states of ratcheting. Science. 2009;325(5943):1014–7. doi: 10.1126/science.1175275 19696352 10.1126/science.1175275 PMC2919209

[5] Kietrys AM, Szopa A, Ba̧kowska-Żywicka K. Structure and function of intersubunit bridges in procaryotic ribosome. Biotechnologia. 2009;115(1):48–58.

[6] Dibrov SM, Parsons J, Hermann T. A model for the study of ligand binding to the ribosomal RNA helix h44. Nucleic Acids Res. 2010;38(13):4458–4465. doi: 10.1093/nar/gkq159 20215440 10.1093/nar/gkq159 PMC2910043

[7] Borovinskaya MA, Pai RD, Zhang W, Schuwirth BS, Holton JM, Hirokawa G, et al Structural basis for aminoglycoside inhibition of bacterial ribosome recycling. Nat Struct Mol Biol. 2007;14(8):727–732. doi: 10.1038/nsmb1271 17660832 10.1038/nsmb1271

[8] Borovinskaya MA, Shoji S, Fredrick K, Cate JHD. Structural basis for hygromycin B inhibition of protein biosynthesis. RNA. 2008;14(8):1590–9. doi: 10.1261/rna.1076908 18567815 10.1261/rna.1076908 PMC2491480

[9] Wilson DN. The A–Z of bacterial translation inhibitors. Crit Rev Biochem Mol Biol. 2009;44(6):393–433. doi: 10.3109/10409230903307311 19929179 10.3109/10409230903307311

[10] Liljas A. Structural aspects of protein synthesis. 2nd ed Singapore: World Scientific Publishing; 2004.

[11] Kulik M, Goral AM, Jasinski M, Dominiak PM, Trylska J. Electrostatic interactions in aminoglycoside-RNA complexes. Biophys J. 2015;108(3):655–665. doi: 10.1016/j.bpj.2014.12.020 25650932 10.1016/j.bpj.2014.12.020 PMC4317553

[12] Garneau-Tsodikova S, Labby KJ. Mechanisms of resistance to aminoglycoside antibiotics: overview and perspectives. Med Chem Commun. 2016;7(1):11–27. doi: 10.1039/C5MD00344J 10.1039/C5MD00344J PMC4752126 26877861

[13] Brodersen DE, Clemons WM, Carter AP, Morgan-Warren RJ, Wimberly BT, Ramakrishnan V. The structural basis for the action of the antibiotics tetracycline, pactamycin, and hygromycin B, on the 30S ribosomal subunit. Cell. 2000;103(7):1143–1154. doi: 10.1016/S0092-8674(00)00216-6 11163189 10.1016/s0092-8674(00)00216-6

[14] Berk V, Zhang W, Pai RD, Cate JHD. Structural basis for mRNA and tRNA positioning on the ribosome. Proc Natl Acad Sci. 2006;103(43):15830–15834. doi: 10.1073/pnas.0607541103 17038497 10.1073/pnas.0607541103 PMC1635088

[15] Zhou J, Lancaster L, Donohue JP, Noller HF. Crystal structures of EF-G-ribosome complexes trapped in intermediate states of translocation. Science. 2013;340(6140):1236086 doi: 10.1126/science.1236086 23812722 10.1126/science.1236086 PMC3979973

[16] Pfister P, Risch M, Brodersen DE, Böttger EC. Role of 16S rRNA helix 44 in ribosomal resistance to hygromycin B. Antimicrob Agents Chemother. 2003;47(5):1496–1502. doi: 10.1128/AAC.47.5.1496-1502.2003 12709313 10.1128/AAC.47.5.1496-1502.2003 PMC153343

[17] Becker B, Cooper MA. Aminoglycoside antibiotics in the 21st century. ACS Chem Biol. 2013;8(1):105–115. doi: 10.1021/cb3005116 23110460 10.1021/cb3005116

[18] Trylska J, Kulik M. Interactions of aminoglycoside antibiotics with rRNA. Biochem Soc Trans. 2016;44(4):987–993. doi: 10.1042/BST20160087 27528743 10.1042/BST20160087

[19] Chandrika NT, Garneau-Tsodikova S. A review of patents (2011–2015) towards combating resistance to and toxicity of aminoglycosides. Med Chem Commun. 2016;7(1):50–68. doi: 10.1039/C5MD00453E 10.1039/C5MD00453E PMC4806794 27019689

[20] Li M, Duc ACE, Klosi E, Pattabiraman S, Spaller MR, Chow CS. Selection of peptides that target the aminoacyl-tRNA site of bacterial 16S ribosomal RNA. Biochemistry. 2009;48(35):8299–8311. doi: 10.1021/bi900982t 19645415 10.1021/bi900982t PMC2752669

[21] Llano-Sotelo B, Klepacki D, Mankin AS. Selection of Small Peptides, Inhibitors of Translation. J Mol Biol. 2009;391(5):813–819. doi: 10.1016/j.jmb.2009.06.069 19576904 10.1016/j.jmb.2009.06.069 PMC2734330

[22] Trylska J, Thoduka SG, Da̧browska Z. Using sequence-specific oligonucleotides to inhibit bacterial rRNA. ACS Chem Biol. 2013;8(6):1101–9. doi: 10.1021/cb400163t 23631412 10.1021/cb400163t

[23] Gupta P, Muse O, Rozners E. Recognition of double-stranded RNA by guanidine-modified peptide nucleic acids. Biochemistry. 2012;51(1):63–73. doi: 10.1021/bi201570a 22146072 10.1021/bi201570a PMC3254705

[24] Zengeya T, Gupta P, Rozners E. Sequence selective recognition of double-stranded RNA using triple helix-forming peptide nucleic acids. Methods Mol Biol. 2014;1050:83–94. doi: 10.1007/978-1-62703-553-8_7 24297352 10.1007/978-1-62703-553-8_7

[25] Hansen J, Platten F, Wagner D, Egelhaaf SU. Tuning protein–protein interactions by cosolvents: specific effects of ionic and non-ionic additives on protein phase behavior. Phys Chem Chem Phys. 2016;18(39):10270–10280. doi: 10.1039/C5CP07285A 27020538 10.1039/c5cp07285a

[26] Abelian A, Walsh AP, Lentzen G, Aboul-Ela F, Gait MJ. Targeting the A site RNA of the Escherichia coli ribosomal 30 S subunit by 2’-O-methyl oligoribonucleotides: a quantitative equilibrium dialysis binding assay and differential effects of aminoglycoside antibiotics. Biochem J. 2004;383(Pt 2):201–8. doi: 10.1042/BJ20040246 15294017 10.1042/BJ20040246 PMC1134060

[27] Thoduka SG, Zaleski PA, Da̧browska Z, Równicki M, Stróżecka J, Górska A, et al Analysis of ribosomal inter-subunit sites as targets for complementary oligonucleotides. Biopolymers. 2017;107(4):e23004 doi: 10.1002/bip.23004 10.1002/bip.23004 27858985

[28] Romanowska J, Setny P, Trylska J. Molecular dynamics study of the ribosomal A-site. J Phys Chem B. 2008;112(47):15227–15243. doi: 10.1021/jp806814s 18973356 10.1021/jp806814s PMC2665305

[29] Panecka J, Šponer J, Trylska J. Conformational dynamics of bacterial and human cytoplasmic models of the ribosomal A-site. Biochimie. 2015;112:96–110. doi: 10.1016/j.biochi.2015.02.021 25748164 10.1016/j.biochi.2015.02.021

[30] Mergny JL, Lacroix L. Analysis of Thermal Melting Curves. Oligonucleotides. 2003;13(6):515–537. doi: 10.1089/154545703322860825 15025917 10.1089/154545703322860825

[31] Puglisi JD, Tinoco I. Absorbance melting curves of RNA. Methods Enzymol. 1989;180:304–325. doi: 10.1016/0076-6879(89)80108-9 2482421 10.1016/0076-6879(89)80108-9

[32] Chaires JB. Possible origin of differences between van’t Hoff and calorimetric enthalpy estimates. In: Biophys. Chem. vol. 64; 1997 p. 15–23. doi: 10.1016/S0301-4622(96)02205-3 9127935 10.1016/s0301-4622(96)02205-3

[33] Freire E, Mayorga OL, Straume M. Isothermal titration calorimetry. Anal Chem. 1990;62(18):950A–959A.

[34] Phillips JC, Braun R, Wang W, Gumbart J, Tajkhorshid E, Villa E, et al Scalable molecular dynamics with NAMD. J Comput Chem. 2005;26:1781–1802. doi: 10.1002/jcc.20289 16222654 10.1002/jcc.20289 PMC2486339

[35] Cornell WD, Cieplak P, Bayly CI, Gould IR, Merz KM, Ferguson DM, et al A second generation force field for the simulation of proteins, nucleic acids, and organic molecules. J Am Chem Soc. 1995;117(19):5179–5197.

[36] Pérez A, Marchán I, Svozil D, Sponer J, Cheatham TE, Laughton CA, et al Refinement of the AMBER Force Field for Nucleic Acids: Improving the Description of *α*/*γ* Conformers. Biophys J. 2007;92(11):3817–3829. doi: 10.1529/biophysj.106.097782 17351000 10.1529/biophysj.106.097782 PMC1868997

[37] Zgarbová M, Otyepka M, Šponer J, Mládek A, Banáš P, Cheatham TE, et al Refinement of the Cornell et al. Nucleic acids force field based on reference quantum chemical calculations of glycosidic torsion profiles. J Chem Theory Comput. 2011;7(9):2886–2902. doi: 10.1021/ct200162x 21921995 10.1021/ct200162x PMC3171997

[38] Martyna GJ, Tobias DJ, Klein ML. Constant pressure molecular dynamics algorithms. J Chem Phys. 1994;101(5):4177–4189. doi: 10.1063/1.467468

[39] Feller SE, Zhang Y, Pastor RW, Brooks BR. Constant pressure molecular dynamics simulation: The Langevin piston method. J Chem Phys. 1995;103(11):4613–4621. doi: 10.1063/1.470648

[40] Ryckaert JP, Ciccotti G, Berendsen HJC. Numerical Integration of the Cartesian Equations of Motion of a System with Constrains: Molecular Dynamics of n-Alkanes. J Comput Phys. 1977;23(3):327–341. doi: 10.1016/0021-9991(77)90098-5

[41] Darden T, Perera L, Li L, Lee P. New tricks for modelers from the crystallography toolkit: The particle mesh Ewald algorithm and its use in nucleic acid simulations. Structure. 1999;7(3):R55—R60. doi: 10.1016/S0969-2126(99)80033-1 10368306 10.1016/s0969-2126(99)80033-1

[42] Hobbie SN, Kalapala SK, Akshay S, Bruell C, Schmidt S, Dabow S, et al Engineering the rRNA decoding site of eukaryotic cytosolic ribosomes in bacteria. Nucleic Acids Res. 2007;35(18):6086–6093. doi: 10.1093/nar/gkm658 17766247 10.1093/nar/gkm658 PMC2094070

[43] Jorgensen WL, Chandrasekhar J, Madura JD, Impey RW, Klein ML. Comparison of simple potential functions for simulating liquid water. J Chem Phys. 1983;79(2):926–935. doi: 10.1063/1.445869

[44] Noy A, Soteras I, Javier Luque F, Orozco M. The impact of monovalent ion force field model in nucleic acids simulations. Phys Chem Chem Phys. 2009;11(45):10596 doi: 10.1039/b912067j 20145804 10.1039/b912067j

[45] Robbins TJ, Wang Y. Effect of initial ion positions on the interactions of monovalent and divalent ions with a DNA duplex as revealed with atomistic molecular dynamics simulations. J Biomol Struct Dyn. 2013;31(11):1311–1323. doi: 10.1080/07391102.2012.732344 23153112 10.1080/07391102.2012.732344

[46] Case DA, Cheatham TE, Darden T, Gohlke H, Luo R, Merz KM, et al The Amber biomolecular simulation programs. J Comput Chem. 2005;26(16):1668–1688. doi: 10.1002/jcc.20290 16200636 10.1002/jcc.20290 PMC1989667

[47] Mura C, McCammon JA. Molecular dynamics of a kappaB DNA element: base flipping via cross-strand intercalative stacking in a microsecond-scale simulation. Nucleic Acids Res. 2008;36(15):4941–55. doi: 10.1093/nar/gkn473 18653524 10.1093/nar/gkn473 PMC2528173

[48] Panecka J, Mura C, Trylska J. Molecular dynamics of potential rRNA binders: Single-stranded nucleic acids and some analogues. J Phys Chem B. 2011;115(3):532–546. doi: 10.1021/jp106404u 21192664 10.1021/jp106404u

[49] Górska A, Jasiński M, Trylska J. MINT: software to identify motifs and short-range interactions in trajectories of nucleic acids. Nucleic Acids Res. 2015;43(17):e114 doi: 10.1093/nar/gkv559 26024667 10.1093/nar/gkv559 PMC4787793

[50] Michaud-Agrawal N, Denning EJ, Woolf TB, Beckstein O. MDAnalysis: A toolkit for the analysis of molecular dynamics simulations. J Comput Chem. 2011;32(10):2319–2327. doi: 10.1002/jcc.21787 21500218 10.1002/jcc.21787 PMC3144279

[51] Leontis NB, Westhof E. Analysis of RNA motifs. Curr Opin Struct Biol. 2003;13(3):300–308. doi: 10.1016/S0959-440X(03)00076-9 12831880 10.1016/s0959-440x(03)00076-9

[52] Song K, Campbell AJ, Bergonzo C, de los Santos C, Grollman AP, Simmerling C. An improved reaction coordinate for nucleic acid base flipping studies. J Chem Theory Comput. 2009;5(11):3105–3113. doi: 10.1021/ct9001575 26609990 10.1021/ct9001575 PMC5442445

[53] Daura X, Gademann K, Jaun B, Seebach D, van Gunsteren WF, Mark AE. Peptide Folding: When Simulation Meets Experiment. Angew Chemie Int Ed. 1999;38(1–2):236–240. doi: 10.1002/(SICI)1521-3773(19990115)38:1/2%3C236::AID-ANIE236%3E3.0.CO;2-M

[54] van der Spoel D, Lindahl E, Hess B, Groenhof G, Mark AE, Berendsen HJC. GROMACS: Fast, Flexible, and Free. J Comput Chem. 2005;26:1701–1718. doi: 10.1002/jcc.20291 16211538 10.1002/jcc.20291

[55] Blin G, Denise A, Dulucq S, Herrbach C, Touzet H. Alignments of RNA structures. IEEE/ACM Trans Comput Biol Bioinforma. 2010;7(2):309–322. doi: 10.1109/TCBB.2008.28 10.1109/TCBB.2008.28 20431150

[56] Humphrey W, Dalke A, Schulten K. VMD: Visual molecular dynamics. J Mol Graph. 1996;14(1):33–38. doi: 10.1016/0263-7855(96)00018-5 8744570 10.1016/0263-7855(96)00018-5

[57] Pettersen EF, Goddard TD, Huang CC, Couch GS, Greenblatt DM, Meng EC, et al UCSF Chimera—A Visualization System for Exploratory Research and Analysis. J Comput Chem. 2004;25(13):1605–1612. doi: 10.1002/jcc.20084 15264254 10.1002/jcc.20084

[58] Naghibi H, Tamura A, Sturtevant JM. Significant discrepancies between van’t Hoff and calorimetric enthalpies. Proc Natl Acad Sci. 1995;92(12):5597–5599. doi: 10.1073/pnas.92.12.5597 7777555 10.1073/pnas.92.12.5597 PMC41743

[59] Liu Y, Sturtevant JM. Significant discrepancies between van’t hoff and calorimetric enthalpies. II. Protein Sci. 1995;4(12):2559–2561. doi: 10.1002/pro.5560041212 8580846 10.1002/pro.5560041212 PMC2143031

[60] Liu Y, Sturtevant JM. Significant discrepancies between van’t Hoff and calorimetric enthalpies. III. In: Biophys. Chem. vol. 64 Elsevier; 1997 p. 121–126. doi: 10.1016/S0301-4622(96)02229-6 17029832 10.1016/s0301-4622(96)02229-6

[61] Horn JR, Russell D, Lewis EA, Murphy KP. Van’t Hoff and calorimetric enthalpies from isothermal titration calorimetry: Are there significant discrepancies? Biochemistry. 2001;40(6):1774–1778. 11327839 10.1021/bi002408e

[62] Horn JR, Brandts JF, Murphy KP. Van’t Hoff and calorimetric enthalpies II: Effects of linked equilibria. Biochemistry. 2002;41(23):7501–7507. 12044184 10.1021/bi025626b

[63] Mikulecky PJ, Feig AL. Heat capacity changes associated with DNA duplex formation: Salt- and sequence-dependent effects. Biochemistry. 2006;45(2):604–616. doi: 10.1021/bi0517178 16401089 10.1021/bi0517178 PMC2465463

[64] Mikulecky PJ, Feig AL. Heat capacity changes associated with nucleic acid folding. Biopolymers. 2006;82(1):38–58. doi: 10.1002/bip.20457 16429398 10.1002/bip.20457 PMC2465468

[65] Sharp K. Entropy–enthalpy compensation: Fact or artifact? Protein Sci. 2001;10(3):661–667. doi: 10.1110/ps.37801 11344335 10.1110/ps.37801 PMC2374136

[66] Coimbatore Narayanan B, Westbrook J, Ghosh S, Petrov AI, Sweeney B, Zirbel CL, et al The Nucleic Acid Database: New features and capabilities. Nucleic Acids Res. 2014;42(D1). doi: 10.1093/nar/gkt980 24185695 10.1093/nar/gkt980 PMC3964972

[67] Réblová K, Lankaš F, Rázga F, Krasovska MV, Koča J, Šponer J. Structure, dynamics, and elasticity of free 16S rRNA helix 44 studied by molecular dynamics simulations. Biopolymers. 2006;82(5):504–520. doi: 10.1002/bip.20503 16538608 10.1002/bip.20503

[68] Panecka J, Havrila M, Réblová K, Šponer J, Trylska J. Role of S-turn2 in the structure, dynamics, and function of mitochondrial ribosomal A-site. A bioinformatics and molecular dynamics simulation study. J Phys Chem B. 2014;118(24):6687–6701. doi: 10.1021/jp5030685 24845793 10.1021/jp5030685

[69] Panecka J, Mura C, Trylska J. Interplay of the bacterial ribosomal A-site, S12 protein mutations and paromomycin binding: A molecular dynamics study. PLoS One. 2014;9(11). doi: 10.1371/journal.pone.0111811 25379961 10.1371/journal.pone.0111811 PMC4224418

[70] Flyvbjerg H, Petersen HG. Error estimates on averages of correlated data. J Chem Phys. 1989;91(1):461–466. doi: 10.1063/1.457480

[71] Hess B. Determining the shear viscosity of model liquids from MD simulations. J Chem Phys. 2002;116(1):209–217. doi: 10.1063/1.1421362

[72] Grossfield A, Zuckerman DM. Chapter 2 Quantifying Uncertainty and Sampling Quality in Biomolecular Simulations. Annu Rep Comput Chem. 2009;5:23–48. doi: 10.1016/S1574-1400(09)00502-7 20454547 10.1016/S1574-1400(09)00502-7 PMC2865156

[73] Knapp B, Frantal S, Cibena M, Schreiner W, Bauer P. Is an Intuitive Convergence Definition of Molecular Dynamics Simulations Solely Based on the Root Mean Square Deviation Possible? J Comput Biol. 2011;18(8):997–1005. doi: 10.1089/cmb.2010.0237 21702691 10.1089/cmb.2010.0237 PMC3145956

[74] Behrens S, Rühland C, Inácio J, Huber H, Fonseca Á, Spencer-Martins I, et al In situ accessibility of small-subunit rRNA of members of the domains Bacteria, Archaea, and Eucarya to Cy3-labeled oligonucleotide probes. Appl Environ Microbiol. 2003;69(3):1748–1758. doi: 10.1128/AEM.69.3.1748-1758.2003 12620867 10.1128/AEM.69.3.1748-1758.2003 PMC150112

[75] Wimberly BT, Brodersen DE, Clemons WM, Morgan-Warren RJ, Carter AP, Vonrhein C, et al Structure of the 30S ribosomal subunit. Nature. 2000;407(6802):327–39. doi: 10.1038/35030006 11014182 10.1038/35030006

[76] Schuwirth BS, Borovinskaya MA, Hau CW, Zhang W, Vila-Sanjurjo A, Holton JM, et al Structures of the bacterial ribosome at 3.5 A resolution. Science. 2005;310(5749):827–34. doi: 10.1126/science.1117230 16272117 10.1126/science.1117230

[77] François B, Russell RJM, Murray JB, Aboul-ela F, Masquida B, Vicens Q, et al Crystal structures of complexes between aminoglycosides and decoding A site oligonucleotides: Role of the number of rings and positive charges in the specific binding leading to miscoding. Nucleic Acids Res. 2005;33(17):5677–5690. doi: 10.1093/nar/gki862 16214802 10.1093/nar/gki862 PMC1251667

[78] Murray JB, Meroueh SO, Russell RJM, Lentzen G, Haddad J, Mobashery S. Interactions of designer antibiotics and the bacterial ribosomal aminoacyl-tRNA site. Chem Biol. 2006;13(2):129–138. doi: 10.1016/j.chembiol.2005.11.004 16492561 10.1016/j.chembiol.2005.11.004

[79] Kondo J, Westhof E. The bacterial and mitochondrial ribosomal A-site molecular switches possess different conformational substates. Nucleic Acids Res. 2008;36(8):2654–2666. doi: 10.1093/nar/gkn112 18346970 10.1093/nar/gkn112 PMC2377432

[80] Kaul M, Barbieri CM, Pilch DS. Fluorescence-Based Approach for Detecting and Characterizing Antibiotic-Induced Conformational Changes in Ribosomal RNA: Comparing Aminoglycoside Binding to Prokaryotic and Eukaryotic Ribosomal RNA Sequences. J Am Chem Soc. 2004;126(11):3447–3453. doi: 10.1021/ja030568i 15025471 10.1021/ja030568i

[81] Hussain T, Llácer JL, Wimberly BT, Kieft JS, Ramakrishnan V. Large-Scale Movements of IF3 and tRNA during Bacterial Translation Initiation. Cell. 2016;167(1):133–144.e13 doi: 10.1016/j.cell.2016.08.074 27662086 10.1016/j.cell.2016.08.074 PMC5037330

[82] Buske FA, Mattick JS, Bailey TL. Potential in vivo roles of nucleic acid triple-helices. RNA Biol. 2011;8(3):427–439. doi: 10.4161/rna.8.3.14999 21525785 10.4161/rna.8.3.14999 PMC3218511

[83] Zhou Y, Kierzek E, Loo ZP, Antonio M, Yau YH, Chuah YW, et al Recognition of RNA duplexes by chemically modified triplex-forming oligonucleotides. Nucleic Acids Res. 2013;41(13):6664–6673. doi: 10.1093/nar/gkt352 23658228 10.1093/nar/gkt352 PMC3711454

[84] Šulc P, Ouldridge TE, Romano F, Doye JPK, Louis AA. Modelling toehold-mediated RNA strand displacement. Biophys J. 2015;108(5):1238–47. doi: 10.1016/j.bpj.2015.01.023 25762335 10.1016/j.bpj.2015.01.023 PMC4375624

