# Supplementary figures and images for: Interactions of 2’-O-methyl oligoribonucleotides with the RNA models of the 30S subunit A-site
Source: PLoS One. 2018 Jan 19;13(1):e0191138. doi: 10.1371/journal.pone.0191138 (PMC5774723; doi:10.1371/journal.pone.0191138)

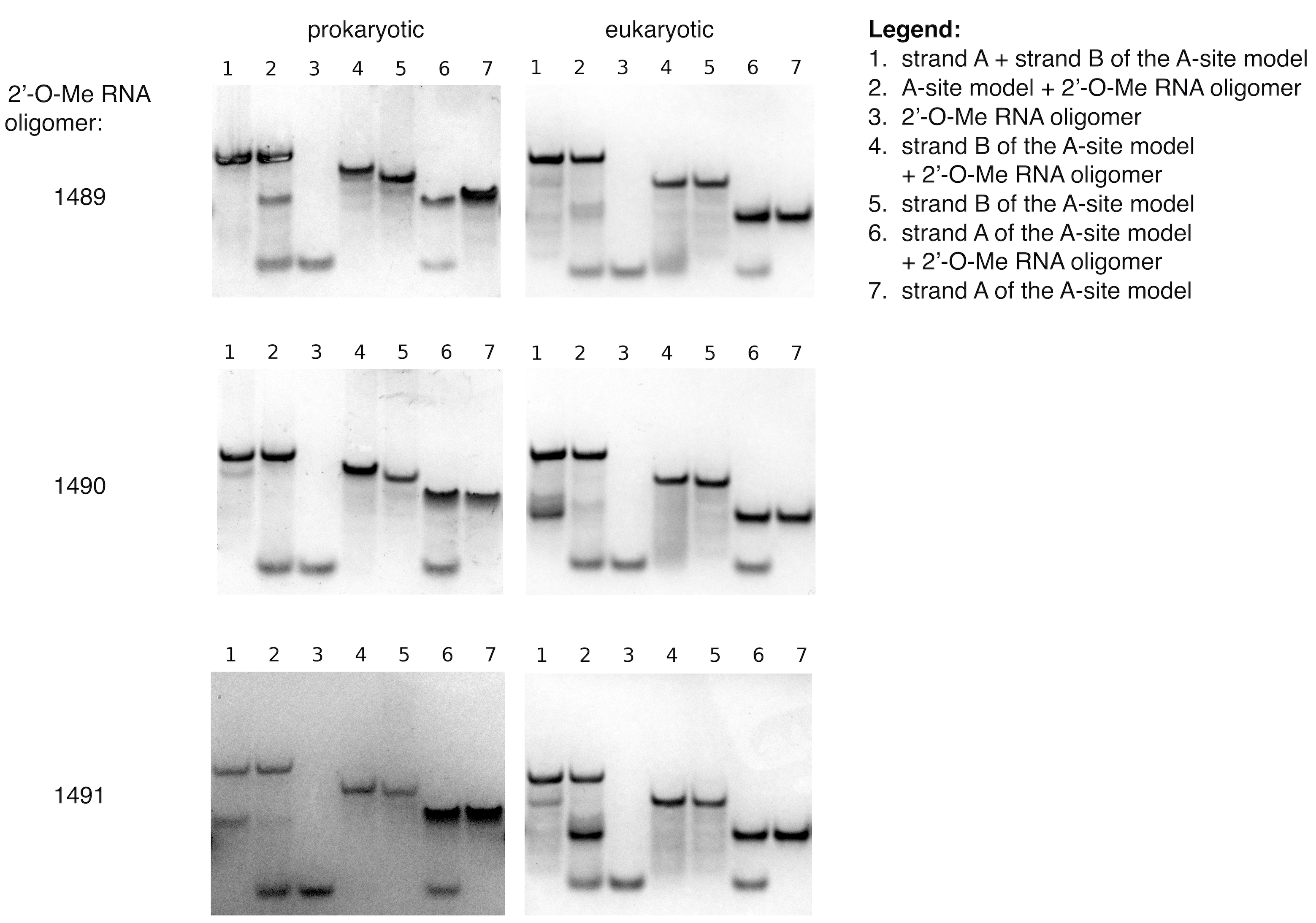

Supplement: S1 Fig — For the rRNA and 2’-O-Me RNA sequences see Fig 2 in the main text. (TIFF) [file pone.0191138.s001.tiff]

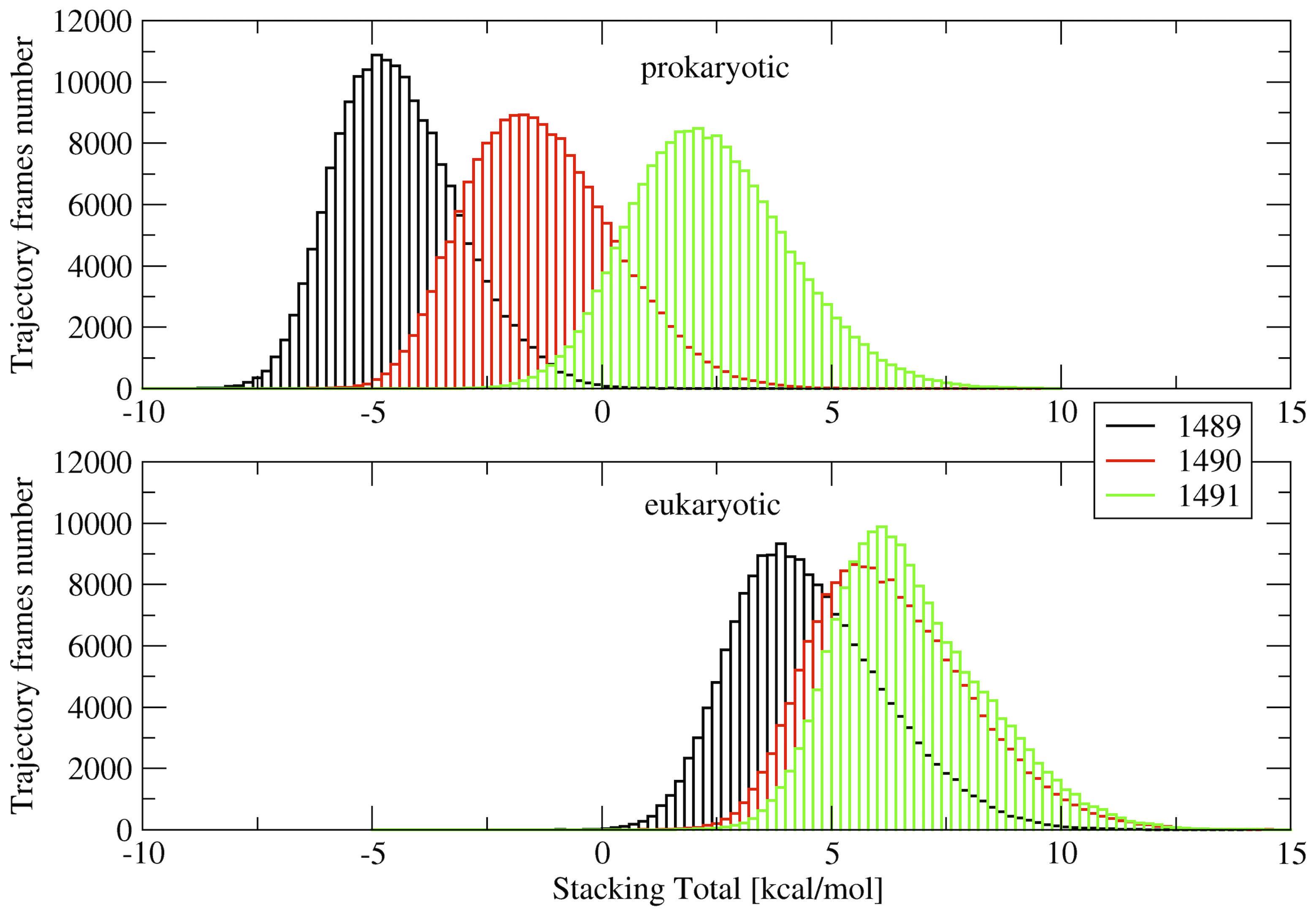

Supplement: S2 Fig — For average and standard deviation values see Table 6 in the main text. (TIFF) [file pone.0191138.s002.tiff]

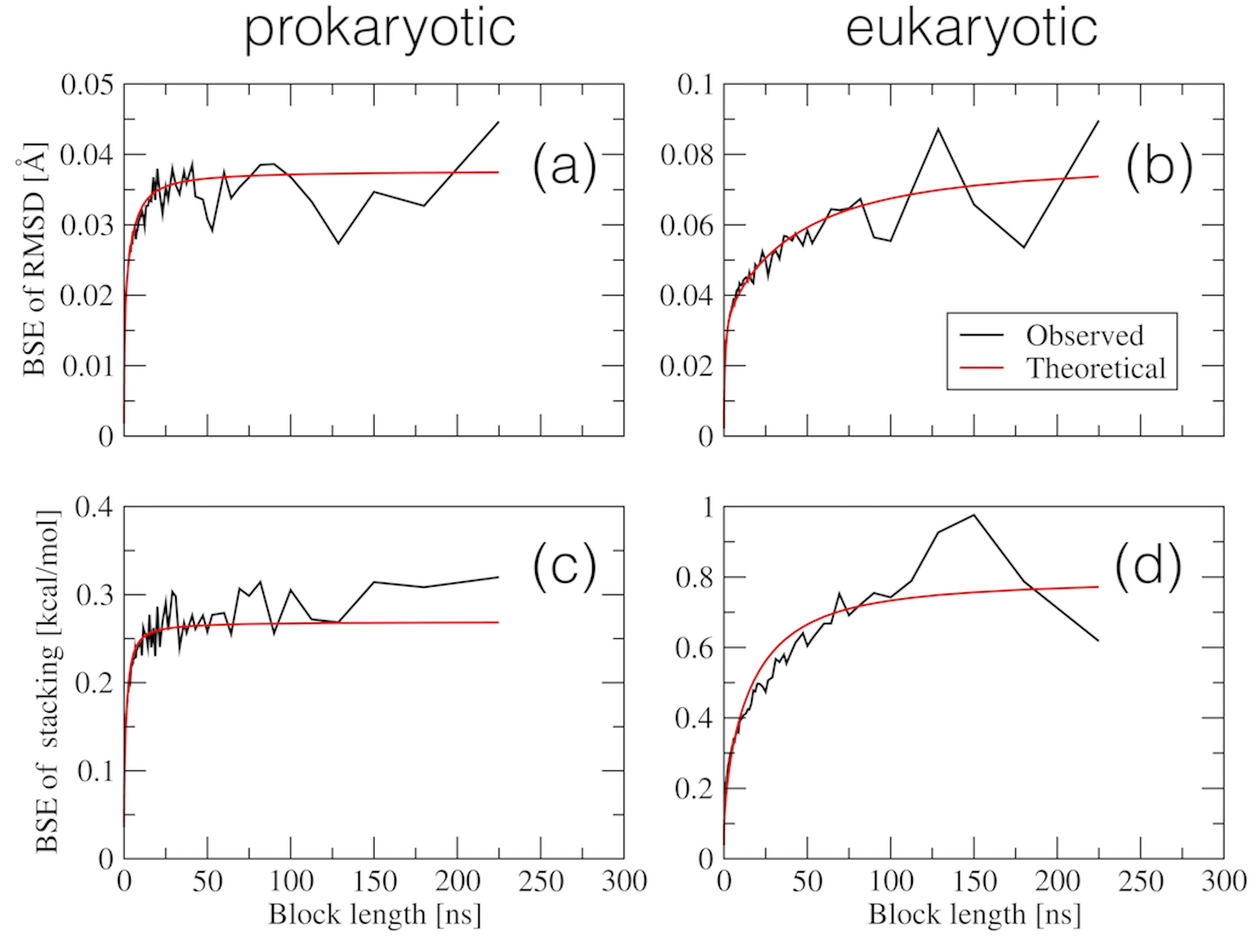

Supplement: S3 Fig — a) RMSD of the prokaryotic model, b) RMSD of the eukaryotic model, c) stacking energy of the prokaryotic model, d) stacking energy of the eukaryotic model. For the time-series of these values see Fig 6a) and 6b) in the main text. The Block Standard Error (BSE) values are plotted as a function of the block size (black line). In addition, the analytical block average curves (red line) are plotted with the assumption that the autocorrelation is a sum of two exponentials (see [71] in the main text for details and complete derivation). (TIF) [file pone.0191138.s003.tif]
